# Variation in Modern Human Deciduous Molar Enamel Formation Time

**DOI:** 10.1002/ajpa.70156

**Published:** 2025-11-14

**Authors:** Patrick Mahoney, Gina McFarlane, Petrina Barnard, Rosie Pitfield, Mackie C. O'Hara, Alfredo Coppa, Carmen Esposito, Alessandra Sperduti, Chris Deter, Alessia Nava, Carolina Loch

**Affiliations:** ^1^ The Histology Lab, School of Natural Sciences, University of Kent Canterbury UK; ^2^ Department of Social Sciences University of Auckland Auckland New Zealand; ^3^ Department of Biology Ball State University Muncie Indiana USA; ^4^ Department of Environmental Biology Sapienza University of Rome Rome Italy; ^5^ Department of Evolutionary Anthropology University of Vienna Vienna Austria; ^6^ Department of Cultural Heritage University of Bologna Ravenna Italy; ^7^ Museum of Civilizations Rome Italy; ^8^ Department of Asian African Mediterranean Studies University of Naples L'Orientale Naples Italy; ^9^ Human Osteology Lab, School of Natural Sciences, University of Kent Canterbury UK; ^10^ Department of Odontostomatological and Maxillofacial Sciences Sapienza University of Rome Rome Italy; ^11^ Sir John Walsh Research Institute, Faculty of Dentistry, University of Otago Dunedin New Zealand

**Keywords:** deciduous teeth, dental development, enamel formation

## Abstract

**Objectives:**

Histologically derived deciduous molar enamel formation times hold great potential for accessing information about the prenatal and postnatal ontogeny of juvenile fossil hominins. Yet our understanding of these formation times in modern humans is limited which inhibits comparative analyses. Here we utilize histology to investigate geographic and temporal variation in prenatal, postnatal, and total enamel cusp formation times among modern humans. We examine some of the processes whereby differences in formation time can arise by considering the underlying rate at which enamel forms and the average amount of enamel that is produced.

**Samples and Methods:**

Longitudinal thin sections of *n* = 356 deciduous first and second deciduous molars were selected from eight populations. Present‐day samples were from the United Kingdom, North America, and Aotearoa New Zealand. Archaeological samples represented the Medieval, Roman, and Iron Age periods. Formation times were calculated from prism lengths and daily cross striations.

**Results:**

Total cusp formation times for present‐day populations were similar except for Pacific Island peoples whose molars formed over a relatively short period. Enamel cusps of the archaeological periods were complete on average 2.5 to 3 months ahead of those from present‐day populations. Longer formation times were due to slower growth rates and an extended period of enamel formation after birth. Enamel thickness varied only slightly between all populations.

**Discussion:**

Our results reveal few differences in formation times between present‐day populations. Enamel formation was complete relatively early in the archaeological samples, which we explore through shifts in the pace of somatic growth.

## Graphical abstract

Human deciduous molar enamel cusp formation times are longer in contemporary populations compared to those from archaeological periods due to slower growth rates and an extended formation period after birth.

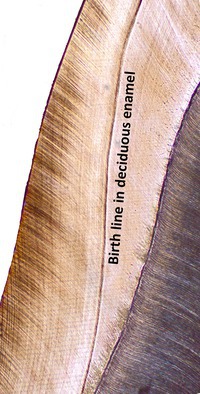

## 1 Introduction

Human enamel forms with a daily rhythm, evidence of which is preserved within teeth as microscopic markings (Schour and Poncher 1937; Massler et al. 1941; Okada 1943; Shinoda 1984; Bromage 1991; Zheng et al. 2013, 2014). These markings can be accessed through histology to reconstruct enamel cusp formation times (Cu‐Ft) when the chronological age of an individual is not available (Boyde 1963, 1964). Most of what is known about formation times calculated in this way comes from studies of permanent teeth (e.g., Reid et al. 1998; Reid and Dean 2000, 2006; Mahoney 2008) that are often incorporated into research that investigates the ontogeny and life history of hominoids (e.g., Bromage and Dean 1985; Beynon et al. 1998; Dean et al. 2001).

Little is known about histologically derived enamel Cu‐Fts of human deciduous molars from present‐day populations (mid‐20th Century AD to present) (Birch and Dean 2014). Only a few studies have reported prenatal or postnatal formation times for archaeological samples of deciduous molars (Mahoney 2011, 2012a; Magri et al. 2024) or other deciduous tooth types (Mahoney 2012b; Nava, Bondioli, et al. 2017; Dean et al. 2020; Peripoli et al. 2023; Martirosyan et al. 2024). Yet, recent comparative studies report that variation in deciduous enamel Cu‐Ft before and after birth, interpreted within a framework that considers the underlying enamel growth rates, can provide unique insights into the ontogeny of juvenile hominins (Nava et al. 2020; Mahoney et al. 2021; Higgins et al. 2024). For example, the deciduous incisor enamel of Krapina Neanderthals forms rapidly in utero and over a brief period after birth, which produces a relatively fast pace of dental development compared to present‐day human juveniles (Mahoney et al. 2021). Other enamel growth mechanisms have been reported for the Baka pygmies, whereby their early postnatal eruption of deciduous canines is facilitated by an extended period of prenatal enamel growth (Tiwa et al. 2024). This renewed interest in deciduous enamel growth is augmented by recent studies of enamel chemistry in relation to nursing, mobility and the distribution of trace elements (Nava et al. 2020; Higgins et al. 2024; Martirosyan et al. 2025), as well as research into the morphology of the birth (neonatal) line (Behie and Miszkiewicz 2019; Hassett et al. 2020; Sipovac et al. 2023), and the increasing number of recovered fossil hominin deciduous teeth (Bolter and Zipfel 2025; Bolter et al. 2025).

### 1.1 Study Aims

Here we build on our previous histology research that examined enamel growth in deciduous teeth from present‐day children (McFarlane et al. 2021; Mahoney et al. 2022; Barnard et al. 2024). Our goal is to identify variation in histologically derived enamel Cu‐Ft amongst modern human deciduous first (dm1) and second (dm2) molars. There are three aims. We compare prenatal, postnatal and total enamel formation times to (1) identify differences in Cu‐Ft between present‐day populations. Present‐day populations are then compared to archaeological samples to (2) identify temporal variation. Our final aim is to (3) gain an understanding of how variation in formation time might arise by considering the rate enamel forms, and the average amount of enamel that is produced. As part of our methodology, and to facilitate comparisons, we develop a practical method to scale the lateral formation time of deciduous molars in relation to the pace change that occurs as cusps gain height.

### 1.2 Current Understanding of Deciduous Enamel Formation Time

#### 1.2.1 Postnatal Formation Times Amongst Present‐Day Populations

There are several approaches available for calculating deciduous molar enamel formation time, including histology, radiographs, and other methods such as measurements of tooth length. Histologically derived enamel Cu‐Fts of deciduous molars from present‐day populations are scarce and geographic variation has not been assessed. Birch and Dean (2014) reported regression formulae to estimate formation time from enamel thickness based upon counts of cross striations in *n* = 4 mandibular dm_1_ and *n* = 4 dm_2_. There are no histologically derived formation times for present‐day maxillary deciduous molars.

A few histology studies of permanent molars have explored variation in total and post‐natal formation times amongst present‐day populations of different ethnicities from different geographic regions. Reid and Dean (2006) observed limited variation in the timing of enamel completion for permanent molar cusps amongst samples drawn from different geographic regions represented by Northern Europe and North America. This differed from their finding for anterior teeth and premolars of South Africans, which formed over shorter periods compared to the same tooth types from Northern Europe (Reid and Dean 2006; Reid et al. 2008). Whether deciduous molars from North American and Northern European populations form over analogous periods has not been determined.

Several radiographic studies have presented the postnatal chronological age at which a developing deciduous molar attains stages of enamel formation (e.g., crown 50% complete, 75% complete) (Gleiser and Hunt Jr. 1955; Moorrees et al. 1963; Gustafson and Koch 1974; Liversidge et al. 1999; AlQahtani et al. 2010; Irurita et al. 2014). Among these, Irurita et al. (2014) observed that the age at crown completion for a mid‐20th century AD Spanish sample was higher compared to the data of Moorrees et al. (1963) from children in the USA, but was similar to formation times presented by Liversidge and Molleson (2004) for a mixed archaeological and present‐day sample from Britain.

Using radiographic methods to score dental age from permanent teeth, Petelo (2011) reported that Polynesian children (Polynesia is a subregion of the Pacific islands) were relatively advanced compared to New Zealand (NZ) children and NZ Māori children. Others observed a gradient in the schedule of dental development whereby the permanent mandibular molar formation of Pacific Island peoples was ahead of Māori, and in turn, Māori were advanced relative to NZ‐European children (Te Moananui et al. 2008). A more recent study reconstructing the dental age of permanent mandibular teeth reported results that supported those of Te Moananui et al. (2008), whereby Pacific Island peoples matured faster than European children (Baylis et al. 2024). Whether the relatively advanced permanent dental development of Pacific Island peoples is present in their deciduous molars has not been assessed.

It is important to note that radiographic and histologically derived enamel formation times are not directly comparable (Beynon et al. 1991). The early and latest stages of enamel mineralization are difficult to detect on radiographs, as they are often not radiopaque leading to relatively shorter formation times (Liversidge 2016). These differences in formation times, between radiographs and histology, have been reported for deciduous molars (Mahoney 2011; Irurita et al. 2014), deciduous canines (Liversidge and Molleson 2004) and permanent teeth (Reid and Dean 2006). This is not a concern when the aim is to relate a mineralization stage to a known chronological age, which is typical of many longitudinal radiographic studies. This can differ from the aim of histology studies where chronological age is often not available, but the earliest and latest stages of enamel mineralization can be accessed and incorporated into formation times (e.g., FitzGerald and Hillson 2009; Mahoney 2011, 2015; Dean et al. 2020; Peripoli et al. 2023; Martirosyan et al. 2024).

#### 1.2.2 Prenatal Formation Times Amongst Present‐Day Populations

Early research reported the degree of mineralization in prenatal deciduous enamel (Mellanby 1927; Kraus and Jordan 1965), the morphology of the neonatal line (Schour 1936) or the extent of deciduous dental development at birth (Logan and Kronfeld 1933). Massler et al. (1941) conducted a pioneering histological study that included deciduous teeth from present‐day populations and reported calcification of first molars in the fifth month in utero, and in the sixth month for deciduous second molars.

More recent studies of prenatal formation times focus on histology as there are ethical considerations when taking radiographs of infants to investigate the prenatal initiation of deciduous teeth (Dean et al. 2020). Histology calculates initiation times retrospectively using incremental markings. Sunderland et al. (1987) reported the earliest onset of dentin mineralization for dm1 in gestational Weeks 16 and 19, and in Weeks 20 to 22 for dm2 (Sunderland et al. 1987). Birch and Dean (2014) used regression equations applied to *n* = 10 thin sections of present‐day dm1 and dm2 to calculate 95% confidence intervals (CI). They reported a CI of 146 to 135 days before birth for the initiation of dm1, and a CI of 122 to 113 days for dm2 (Birch and Dean 2014).

Histologically derived variation in prenatal enamel formation times amongst modern human populations is poorly understood. Tiwa et al. (2024) reported a greater proportion of deciduous canine enamel was formed by birth in Baka pygmies when compared to the prenatal formation times reported by Dean et al. (2020) for a cohort of children from the United Kingdom. The precocious prenatal dental development of the Baka pygmies facilitates an earlier age at tooth emergence, when compared to other African populations (Ramirez Rozzi 2016).

#### 1.2.3 Formation Times in Archaeological Populations

Histology research has focused on archaeological samples from the United Kingdom and Italy (e.g., Mahoney 2011, 2015; Magri et al. 2024). Charts depicting the timing of fractional stages of enamel formation have been published for a small mixed sample of Bronze Age and Medieval deciduous mandibular protoconid cusps of dm_1_ (*n* = 4) and dm_2_ (*n* = 4) from the United Kingdom (Mahoney 2011). Magri et al. (2024) calculated total Cu‐Ft and prenatal formation times (wear stage 1) for a sample of dm_1_ (*n* = 7) and dm^1^ (*n* = 5) from Medieval Italy. Mahoney (2015) reported total Cu‐Ft and prenatal formation time for protocone cusps of Medieval maxillary dm^1^ (*n* = 17) and dm^2^ (*n* = 14) from the United Kingdom.

#### 1.2.4 Temporal Variation in Formation Time

There is limited knowledge about the deciduous dental development of present‐day children compared to those from past populations (Liversidge and Molleson 2004). Those studies that have been undertaken have focused on more recent historical collections for which chronological age was known (Saunders et al. 1993; Cardoso 2007). No study has compared histologically derived deciduous molar Cu‐Ft between present‐day and archaeological samples with the specific aim of identifying temporal variation.

There are good reasons to suspect temporal variation in formation times between present‐day and archaeological populations that relate to the rate at which enamel is secreted, and enamel thickness. Enamel daily secretion rates (DSRs) can be accelerated amongst archaeological samples of deciduous teeth compared to modern samples (Mahoney 2015; Nava, Bondioli, et al. 2017; Nava, Coppa, et al. 2017; Dean et al. 2020; McFarlane et al. 2021). Whether these faster enamel growth rates in more ancient periods relate to shortened Cu‐Ft for deciduous molars has not been assessed. Nava, Bondioli, et al. (2017) observed the daily rate at which enamel gained thickness in deciduous central incisors from the Roman period (100–200 ad) was accelerated compared to modern‐day samples. McFarlane et al. (2021) reported DSRs for a global sample of deciduous molars from present‐day populations (Aotearoa New Zealand, United Kingdom, Canada, France, and Sweden) and noted these rates were on average slower than those reported for a small mixed sample of Bronze Age and Medieval deciduous molars from the United Kingdom. Dean et al. (2020) observed *n* = 6 deciduous canines from present‐day UK children gained height at about half the rate that was reported by Mahoney (2015) for deciduous canines from Medieval United Kingdom. Equivalent results have been presented in studies of permanent teeth as well. Permanent molars and incisors formed at a significantly faster pace during the Roman, Anglo‐Saxon, and Medieval periods in the United Kingdom compared to modern‐day samples (Aris, Mahoney, and Deter 2020; Aris, Mahoney, O'Hara, and Deter 2020).

Enamel thickness is determined by the number of active ameloblasts, their secretory life span, and the total period over which these cells are active (Grine and Martin 1998). Longer periods of ameloblast cell activity can be represented by longer formation times that link to thicker enamel, which has been noted in comparisons of cuspal enamel from present‐day European and South African permanent premolars (Reid et al. 2008; O'Hara 2021). Several factors can influence this relationship and key amongst these is the rate at which enamel forms. For example, interspecific research of fossil hominins observed faster DSRs formed permanent molar enamel crowns with relatively thickened enamel over the same periods as hominin species with thinner enamel that utilized slower DSRs (Lacruz and Bromage 2006; Lacruz et al. 2008).

Enamel thickness may change over relatively short time periods due to the selective forces that act upon tooth surfaces in response to dietary change (e.g., Hlusko 2004). Studies of European archaeological samples of human permanent molars provide support for this proposal (Le Luyer and Bayle 2017; Aris, Mahoney, O'Hara, and Deter 2020). Thickened enamel regions in permanent molar crowns have been related to increased wear from contaminated foods, in comparisons of Neolithic and Mesolithic samples from France, and a similar situation has been proposed for the Early compared to Late Anglo‐Saxon and Medieval periods in the United Kingdom (Le Luyer and Bayle 2017; Aris, Mahoney, O'Hara, and Deter 2020). Whether equivalent temporal variation in enamel thickness is present in archaeological samples of deciduous molars has not been determined.

### 1.3 Predictions

#### 1.3.1 Amongst Present‐Day Populations

If enamel thickness and secretion rates remain generally constant then we do not expect deciduous molar Cu‐Ft to vary between populations from the United Kingdom and North America based upon the similarity in the formation times of permanent molar cusps from Northern European and North American populations (Reid and Dean 2006).

We predict deciduous molar cusps of Māori and Pacific Island peoples should form over a shorter period compared to those of NZ European ethnicity based upon reported differences in permanent mandibular molar formation times (Te Moananui et al. 2008). Alternatively, deciduous molars of Māori and Pacific Island people might have an extended period of enamel growth before birth to facilitate an advanced pace of post‐natal dental development, similar to that observed for the deciduous canines of Baka pygmies (Tiwa et al. 2024). In this scenario, the total period over which deciduous molar enamel forms will be similar among the three NZ populations, but the amount of enamel that forms before birth will be greater among Māori and Pacific Island peoples compared to NZ Europeans.

#### 1.3.2 Between Present‐Day and Archaeological Populations

Diet in the Medieval, Roman, and Iron Age periods of Europe could include an emphasis on grain and cereal foods with varying amounts of meat and fish depending upon factors such as status and seasonal availability (e.g., Dyer 1989, 2000; Hanawalt 1995; Martinez‐Labarga et al. 2009; Woolgar 2010; Farese et al. 2024). Traditional milling methods (Farmer 1992; Keller 1989) can introduce abrasive residue into cereal foods, which is a source of tooth wear (Peters 1982; Lucas et al. 2013; Teaford and Lytle 1996). If this type of abrasive wear elicited an adaptive response, through increased enamel thickness, then we expect the enamel thickness of deciduous molars from these three archaeological periods will be greater compared to present‐day populations from the United Kingdom, whose diet can include processed and softer foods that contain relatively fewer abrasive contaminants. Under these circumstances, we predict that the thicker enamel of the archaeological periods will have a longer Cu‐Ft compared to those from the present day, assuming the enamel growth rates remain generally consistent.

Alternatively, lateral regions of permanent first molar crowns were thinner, and mean DSRs from deciduous central incisors were faster in the Roman period, when compared to present‐day populations in the United Kingdom (Nava, Bondioli, et al. 2017; Aris, Mahoney, O'Hara, and Deter 2020). If deciduous molars from the Roman period also have accelerated DSRs, and a thinner‐enameled crown, then we predict that this will relate to a shortened Cu‐Ft, when compared to deciduous molars from present‐day populations.

When just the three archaeological periods are considered, we predict a temporal shortening in formation times related to a change in enamel secretion rates that will be broadly equivalent to that reported for permanent first molars from the United Kingdom (Aris, Mahoney, O'Hara, and Deter 2020). If deciduous molar enamel thickness remains constant between the Medieval, Roman and Iron Age populations, but enamel DSRs decrease chronologically, then the deciduous molars of the more recent Medieval period will have relatively slow DSRs and a longer formation time compared to the Roman period. Deciduous molars from the Roman period will have a longer Cu‐Ft than those from the Iron Age. The Iron Age will have the shortest Cu‐Ft and fastest DSRs.

## 2 Materials and Methods

Present day samples were from populations in Aotearoa New Zealand (Māori, NZ‐European, and Pacific Island peoples), the United Kingdom and North America (Canada). Archaeological samples were selected from the United Kingdom and Italy to represent a time series (Present day; Medieval 11th to 16th century AD; Roman 1st to 4th century AD; Iron Age 4th to 11th century BC).

### 2.1 Samples, Ethics and Community Consultation

Table 1 provides a list of the 356 samples subdivided by tooth type for the five present‐ day populations (*n* = 216) and three archaeological periods (*n* = 140). Total formation times for *n* = 333 were recorded for this study, and these were supplemented with *n* = 23 Medieval samples taken from Mahoney (2015). The *n* = 23 total cusp formation times in Mahoney (2015) were recorded using the same methodology (prism lengths and secretion rates) as the present study. Chronological age related to tooth formation stage was not known for any sample.

**TABLE 1:** Samples subdivided by tooth type and population.

Population	Lower dm1	Upper dm1	Lower dm2	Upper dm2	Total
Modern					
NZ European	11	11	16	20	58
British	14	12	11	22	59
Māori	10	14	10	11	45
Pacific Island	8	—	8	11	27
Canadian	—	11	6	10	27
Archaeological					
Medieval	15	17	18	14	64
Roman	28	5	6	5	44
Iron age	10	—	6	16	32
Total	96	70	81	109	356

Formation times in this study were recorded from existing and new thin sections. Existing sections of present‐day molars were from previous studies (McFarlane et al. 2021; Mahoney et al. 2022; Barnard et al. 2024). Existing thin sections of Medieval molars (Mahoney 2011, 2013, 2015; Pitfield 2019; Pitfield et al. 2019) and Roman molars were re‐examined for this project. New thin sections of Iron Age samples were produced for this study (see Section 2.2).

Present‐day sections were from over *n* = 1000 clinically extracted and naturally exfoliated deciduous molars (see below). Sections were selected for this project if they had no wear, or a slight amount of cuspal tooth wear, either stage 1 or 2 following Molnar (1971), requiring only slight reconstruction of cuspal enamel. Most of the *n* = 140 archaeological samples were unworn samples that were un‐erupted or had just erupted. Even without reconstruction, slight wear has no effect on the postnatal formation times of deciduous first molars that are calculated from the neonatal line that is preserved in lateral enamel. The line is typically present in the cuspal enamel of second molars as well, beneath the cusp tip, so again, slight wear will not affect the calculation of postnatal formation time.

Māori, NZ‐European, and Pacific Island samples were part of the Biorhythm of Childhood Growth project which was a prospective cohort study investigating childhood development in Aotearoa New Zealand (McFarlane et al. 2021; Mahoney et al. 2022; Floyd et al. 2023; Barnard et al. 2024). Individuals were assigned to Māori, NZ‐European, and Pacific Island ethnicity based upon self‐identification. These anonymized naturally exfoliated and clinically extracted deciduous molars were collected in schools within the city of Dunedin, and by dental and oral health therapists from community oral health clinics of the Whanganui and Hawkes Bay District Health Boards (New Zealand). Ethical approval for histological analysis of these deciduous teeth was obtained from the University of Otago Human Ethics Committee (approval number H19/030). In New Zealand, consultation with Māori is mandated in all areas of research that involve people of Māori descent. Research consultation and approval for sample collection and producing thin sections of Māori deciduous molars were obtained from the Ngāi Tahu Research Consultation Committee.

British samples were from the Kent‐UCL collection and the Percy Butler collection. These were anonymized dental extractions or naturally exfoliated teeth collected during routine dental treatment in the United Kingdom during the 1960s and 1970s. Unlike our NZ samples, these British samples were not subdivided by ethnic (or cultural) groups, and presumably encompassed a broad range of backgrounds. Ethical approval for histological analysis was obtained from the UK National Health Service research ethics committee (REC reference: 19/REM/0126, ID 261173).

Canadian dental samples were anonymized molars collected with consent during the 1970s at Simon Fraser University. Ethical approval for histological analysis was obtained from the UK National Health Service research ethics committee (REC reference: 19/REM/0126, ID 261173).

Medieval samples were from the cemetery of St Gregory's Priory which dates to the 11th to 16th Century AD in Canterbury, England. Formation times for lower dm1 and dm2 were produced for this study. Formation times for upper dm1 and dm2 were recalculated from Mahoney (2015). Historical textual records indicate that the cemetery served the local community, people who could not afford burial fees, as well as adult patients from nearby St John's hospital (Brent 1879; Somner 1703). The site was excavated between 1988 and 1991 (Hicks and Hicks 2001).

Roman samples were from Isola Sacra, which is located about 23 km south‐west of Rome in the region of Lazio, in Italy. The necropolis was used to bury the inhabitants of Portus Romae (the port of Rome) in the 1st to 4th centuries CE. The port was an important trading center linking the Empire with its distant provinces (Garnsey 1999). The people buried at Isola Sacra were middle‐class administrators, traders, merchants, and seafarers, often descended from slaves (Garnsey 1999; Baldassarre et al. 1996). The necropolis has been excavated since the 1920s and 1930s, and to date has yielded more than 2000 individuals whose remains are curated at the Museo delle Civiltà in Rome.

Iron Age samples were from nine archaeological sites (Alfedena, Scurcola Marsicana, La Cona, Capestrano, Caporciano, Mossa, Bithia SA Colonia, Veio Quattro Fontanili, Fontanaccia) in Italy. The samples from these sites were combined to increase the sample size for the Iron Age period. The site of Alfedena (Abruzzo, 6th to 4th century BCE) is located amongst the Apennine mountains of central Italy and is characterized by burials of male warriors. Several studies have investigated this Iron Age community through bioarchaeology, aDNA, and bulk radiogenic isotope analyses (e.g., Bondioli et al. 1986; Sparacello et al. 2011). The funerary site of La Cona is located on the top of an alluvial terrace about 3 km south‐west of Teramo (Abruzzo, Italy). The necropolis covers a chronological span dating from the 8th–6th century BCE. The Scurcola Marsicana necropolis was identified in 1983, along the Imele river (L'Aquila, Abruzzo, Italy) (Colucci and Irti 1983). The necropolis is characterized by tumulus graves, covering a chronological span from the 8th–6th century BCE that is generally attributed to the Sabine population. The necropolis of Capestrano (6th to 4th century BCE) and Caporciano (4th to 1st century BCE) are located near L'Aquila (Abruzzo, Italy) and have been excavated from the 1930s (d'Ercole et al. 2018). Veio is a Villanovan‐Etruscan site located in Tyrrhenian Central Italy that was established from at least the 9th–7th century BCE, and perhaps as early as the 10th–9th BCE (Pacciarelli 2001; Biagi 2019; di Gennaro 2019). During the 8th–7th BCE, Veio became a focal point for long‐distance trade (Bartoloni et al. 2019), evidenced by numerous recovered Levantine and Greek artifacts (Boitani 2019; Cerasuolo 2019).

The necropolis of Quattro Fontanili of Veio consists of *n* = 651 cremations and inhumations, and has been extensively excavated and published (e.g., Ward‐Perkins et al. 1965, Ward‐Perkins et al. 1967; Ward‐Perkins and Falconi Amorelli 1970; Bedello and Fabbricotti 1975). The site of Fermo in the Marche region exemplifies the Villanovan/Proto‐Etruscan expansion within the Italian peninsula (Naso 2000; Esposito et al. 2023; Miranda and Esposito 2021). Fermo is distinguished by a cultural physiognomy of its own, manifested in its topographical location and funerary rituals that are similar to those documented in southern Etruria (Esposito et al. 2023). The Mossa and Misericordia necropolises were positioned on the northern slopes of Girfalco. Recent chronological dates assign these necropolises to the 9th to early 5th centuries BCE (Miranda and Esposito 2021).

Fontanaccia is a small necropolis in central Italy. It is located in the municipality of Barbarano Romano (Viterbo, Lazio) and is dated to an advanced phase of the early Iron Age (Gazzetti and Ghini 2006). The Fontanaccia skeletal remains belong to the Villanovan culture (11th–8th century BCE). Bithia SA Colonia samples were collected as part of an emergency intervention following a sea storm at Chia, in the municipality of Domus de Mariae (Cagliari, Sardinia, Italy), during which several infant inhumation burials were unearthed and recovered. The infants were placed in amphorae (enchytrismòs) and refer to the Phoenician‐Punic site of Bithia (Bartoloni et al. 1996).

### 2.2 Thin Sections

Standard methods were followed to produce histological thin sections. Thin sections of samples from the United Kingdom, Canada, Aotearoa New Zealand, Medieval and Iron Age periods, had been, or were, processed in the Histology Lab, University of Kent. Roman samples were processed in the Histology Lab, Sapienza University of Rome. All thin sections were produced using the same standard methodology. Deciduous molars were embedded in resin (Buehler EpoxiCure) and the protoconids (lower molars) and protocones (upper molars) were selected for sectioning as these are the first‐formed deciduous molar cusps. The section plane traveled through the long axis of each tooth, from the tip of the cusps and dentin horn capturing the mandibular protoconid and maxillary protocone, using a Buehler isomet 4000 precision saw. Sections were fixed to glass microscope slides (Evo Stick resin), ground to a thickness of 70–100 μm (grit P400, P600, P1200), polished with a 0.3 μm aluminium oxide powder (Buehler Micro‐Polish II), cleaned in an ultrasonic bath, dehydrated in 95%–100% ethanol, cleared (Histoclear), and mounted with a coverslip (DPX). Sections were examined using a high‐resolution microscope (Olympus BX53) and digital microscope camera (Olympus DP28). Images were obtained and analyzed in CELL Live Biology imaging software. Oblique sections were identified and removed. Oblique sections are easily identified from the morphology of the dentin horn and slope of the enamel buccal and lingual surfaces. Histology data were produced by PM, GM, and RP.

### 2.3 Histology Data Collection

#### 2.3.1 Enamel Daily Enamel Secretion Rates

**FIGURE 1:**
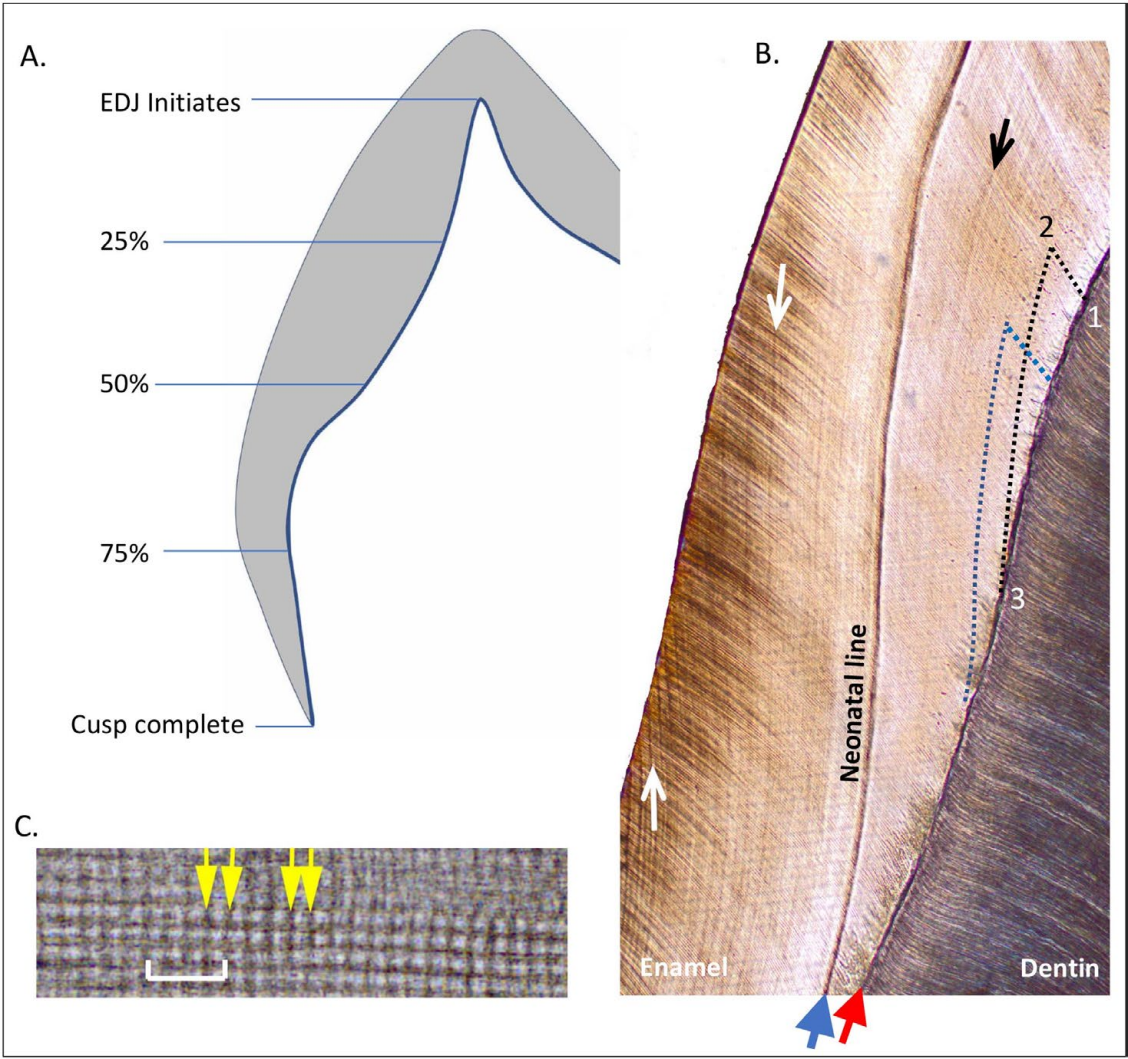
Deciduous molar enamel cusp. (A) Schematic representation of the cusp divided into four segments with each segment representing 25% of the cusp EDJ height. (B) Histological thin section through a present‐day deciduous cusp. Blue arrow points to the neonatal line imaged at magnification of 4× and red arrow points to the enamel‐dentin junction (EDJ). The black arrow to the right of the neonatal line points to a faint prenatal growth marking. The white arrows to the left are postnatal accentuated markings and Retzius lines. Locations 1, 2 and 3 on the EDJ are utilized in the enamel extension calculation (see text for methodology). (C) Enamel cross striations from the mid‐enamel region of a Medieval deciduous cusp, imaged at a magnification of 40×. Cross striations, indicated by yellow arrows, are used to calculate enamel daily secretion rates. Distance between two adjacent striations is equal to 24 h. Prisms run left to right. White scale bar is 17 μm.

Enamel growth is temporarily interrupted by a daily circadian cycle (Lacruz et al. 2012; Zheng et al. 2013, 2014; Papakyrikos et al. 2020). The cycle produces markings that are preserved as cross striations along enamel prisms (Boyde 1989). The distance between adjacent pairs of cross striations is the amount of new enamel deposited over a 24‐h period (Figure 1). This is expressed as an enamel DSR that is measured in μm per day (e.g., Schour and Poncher 1937; Reid et al. 1998). The DSR is a measure of the rate at which developing enamel increases in thickness. These rates are reported here for the archaeological samples; McFarlane et al. (2021) have reported DSRs for a global sample of present‐day deciduous molars.

Daily secretion rates were calculated in cuspal enamel following standard methods (e.g., Beynon et al. 1991). This enamel was subdivided into inner, mid and outer regions, and DSRs were recorded within those regions. Prism lengths corresponding to five consecutive days of enamel secretion were measured in ten locations within each region and used to calculate an overall mean DSR value for that region. For the inner enamel region this included rates near the EDJ, which contributed to the calculation of one final mean value for the inner one‐third of enamel thickness.

In addition to the standard mean DSR values calculated for inner, mid and outer cuspal enamel, a more detailed trajectory of enamel secretion was calculated in the present study for Iron Age dm2 (upper and lower combined). These samples were selected because cross striations were very well preserved in this region. This additional analysis of DSRs was undertaken so that the first formed enamel adjacent to the EDJ could be compared to enamel that formed subsequently over an approximately 60‐day period. A distance of 250 μm was measured along prisms in prenatal cuspal enamel that extended from the EDJ towards the outer surface (approximately the first 60 days of enamel secretion). This distance was subdivided into sequential regions each measuring 25 μm, and cross striations were used to calculate mean secretion rates within those regions.

#### 2.3.2 Enamel Extension Rates

Extension rates measure the speed at which a cusp gains height, and the rate enamel spreads along the forming enamel‐dentin junction (EDJ). The rate is expressed in microns per day. Extension rates are associated with the distribution of surface perikymata (Guatelli‐Steinberg et al. 2012) because secretory ameloblasts initiate at the EDJ and then grow via enamel rods towards the outer enamel surface.

Human molars do not grow in height at a constant rate (Shellis 1984, 1998). Growth commences quickly on the EDJ nearer the cusp tip and then slows towards the end of enamel formation in deciduous molars (Mahoney 2015). Studies of formation time that compare molar lateral surfaces to assess variation in stages of enamel attainment (e.g., 25% of crown height, 50%, 75%) must account for this pace change. One standard way of achieving this is to calculate lateral formation time from Retzius lines multiplied by their periodicity (see Mahoney et al. 2007: their fig. 1). While this method is suitable for permanent molars (e.g., Reid and Dean 2006), it is typically not suitable for deciduous molars, as Retzius lines are rarely preserved along the entire length of deciduous lateral enamel. An alternative methodology was developed here utilizing extension rates to address the pace change in deciduous molar enamel formation, and to additionally provide information about the rate that cusps gain height. This extension rate methodology is not the same as that used in previous studies where successive rates commenced at the location that the previous accentuated marking intersected the EDJ (Mahoney 2015), or at fixed sequential distances from the dentin horn (Mahoney et al. 2024), or over fixed periods (Dean et al. 2020).

Here, extension rates are used to calculate the time taken to form four equal segments of the EDJ from the dentin horn to the cervix (see Figure 1A). The time taken to form the first segment will be less than the fourth segment due to the pace change in the rate at which the EDJ gains height. Formation time for each of the four segments is then recalculated as a percentage of the total time taken to form the EDJ, from the dentin horn to the cervix. These four percentages, representing four segments of the EDJ, are then reused and applied to the lateral enamel formation times we calculate from standard methods—using prism lengths and DSRs in Section 2.3.3 below—so that we could subdivide this lateral formation time into four stages of attainment (lateral enamel 25% complete, 50%, 75% and 100% complete). Our extension rate methodology does not alter the total Cu‐Ft.

First, an enamel prism was followed for 200 μm from the EDJ (see location 1 in Figure 1B) towards the outer enamel surface (location 2). When possible, the number of cross striations was counted along the prism. More usually, mean DSRs were calculated in 50 μm increments along the prisms. These were used to calculate the time taken to form each increment. The four increments were then summed to provide the total formation time for the 200 μm prism length. Enamel incremental growth or accentuated lines were then followed back to the EDJ (location 3 in Figure 1B). The distance between location 1 and 3 on the EDJ was divided by the time taken to form the 200 μm enamel prism length, which gives the extension rate in microns per day.

Two sequential extension rates were calculated for each of the four EDJ segments to derive a mean value for that segment. In the first segment, an initial rate was calculated commencing at 200 μm away from the dentin horn on the EDJ, and then another rate was calculated at 500 μm away from the horn. In the second segment, a rate was calculated commencing at 25% of the EDJ length, and then another rate was calculated in the same segment 500 μm further down the EDJ. This was repeated for segments three and four so that two extension rates were calculated for each segment.

These extension rates were calculated for a representative sample of the present‐day populations (British, NZ European, Māori and Pacific Island peoples) and the three archaeological periods. There is a minimum of *n* = 5 and a maximum of *n* = 20 for upper and lower dm2 for each population. The aim was to make extension rates calculated for each segment comparable between individuals with different EDJ lengths. Ultimately though, extension rates are independent growth processes meaning there is variation between the same segments from different individuals, which will contribute towards an associated standard deviation.

#### 2.3.3 Molar Enamel Cusp Total Formation Time (Cu‐Ft)

The total molar enamel Cu‐Ft of the mandibular protoconid and maxillary protocone was reconstructed by summing the formation time of the cuspal and lateral enamel regions. Cuspal enamel formation time was calculated by measuring the thickness of enamel from the tip of the dentin horn to the outermost enamel surface following the trajectory of a prism. This distance was divided by the mean DSR from inner, mid and outer enamel areas. A correction factor (enamel thickness × correction factor/mean daily rate of secretion) of 1.05 was used (e.g., Mahoney 2011 for dm1 and dm2; and also see Schwartz et al. 2003). The correction factor accounts for slight changes in prism length resulting from prism decussation (Risnes 1986). The three formation times were summed to give an overall cuspal formation time.

**FIGURE 2:**
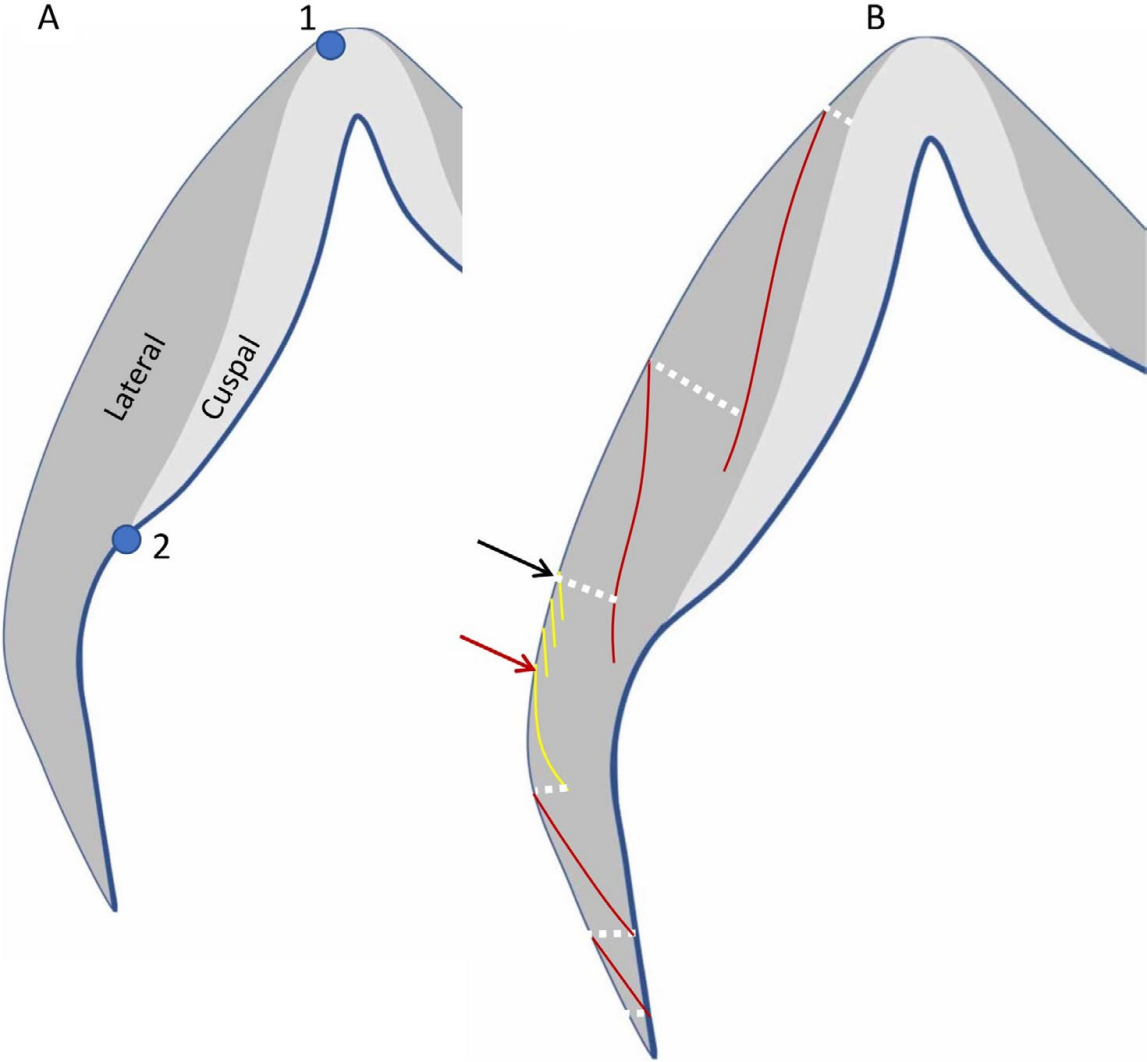
Calculating lateral enamel formation time. (A) Cuspal and lateral enamel regions. (B) Navigating between accentuated incremental lines, and Retzius lines, using prism lengths. See Section 2.3.3 for a description of the methodology.

Lateral enamel formation time was calculated from enamel prism lengths divided by local mean DSRs to navigate between incremental growth lines, accentuated markings, and Retzius lines (Mahoney et al. 2007). Each cusp thin section was subdivided into cuspal and lateral enamel (illustrated in Figure 2A) by following the first Retzius line that emerges onto the outermost enamel surface (location 1 in Figure 2A) back to the EDJ (location 2 in Figure 2A). This line demarcates cuspal and lateral enamel and lies almost parallel to the long axis of the crown, reflecting the relatively faster extension of deciduous relative to permanent teeth (Dean and Wood 1981). In cases where a Retzius line was not present between the cuspal and lateral enamel, then the nearest accentuated marking or clear incremental growth line was used as a template to manually draw the dividing line onto the image using the software CELL. This dividing line represents the mineralising front, and any point along the line, is the same point in time. A prism path indicated by the dashed white line in Figure 2B was followed from the dividing line towards the outermost surface until it intersected with an accentuated marking or clear growth line (indicated by the red line in Figure 2B) at the external surface. The length of the prism was divided by local daily secretion rates to calculate the prism formation time and thus the enamel formation time for that region. This procedure was repeated using the accentuated marking at the enamel surface as the new starting point. Again, any point along that line represents the same point in time. In cases where Retzius lines and cross striations were present together, Retzius periodicity was incorporated into the calculation of lateral formation time. In Figure 2B, four Retzius lines (indicated by the yellow lines between the black and red arrows) have a periodicity of 5 days, meaning this enamel region would form over 15 days (four lines = 3 days). In this example, the fourth Retzius line would then be the starting point for the next prism length measurement. The final calculation of lateral formation time is the sum of all formation times from prism lengths, plus the 15 days calculated from Retzius periodicity.

#### 2.3.4 Pre‐, and Postnatal Enamel Formation Time

Rushton (1933) and Schour (1936) were the first to identify the neonatal line (NNL) in deciduous teeth. In thin sections under transmitted light, the NNL is typically the most prominent dark accentuated line to form in deciduous enamel (FitzGerald and Saunders 2005; Macchiarelli et al. 2006; Humphrey et al. 2008; Sabel et al. 2008; Birch and Dean 2009; Mahoney 2011, 2012a, 2012b, 2015; Kierdorf et al. 2020; Dean et al. 2020; Peripoli et al. 2023; Magri et al. 2024; Martirosyan et al. 2024). The line can sometimes appear as a hypomineralized band (Sabel et al. 2008) or as a discontinuity in prisms (Whittaker and Richards 1978), and can sometimes be matched by a prominent marking in dentin (Mahoney et al. 2021). Not all studies indicate that the NNL is hypomineralized though, but instead report an altered crystallinity with increased levels of the trace element zinc (Dean et al. 2019; Martirosyan et al. 2025).

**FIGURE 3:**
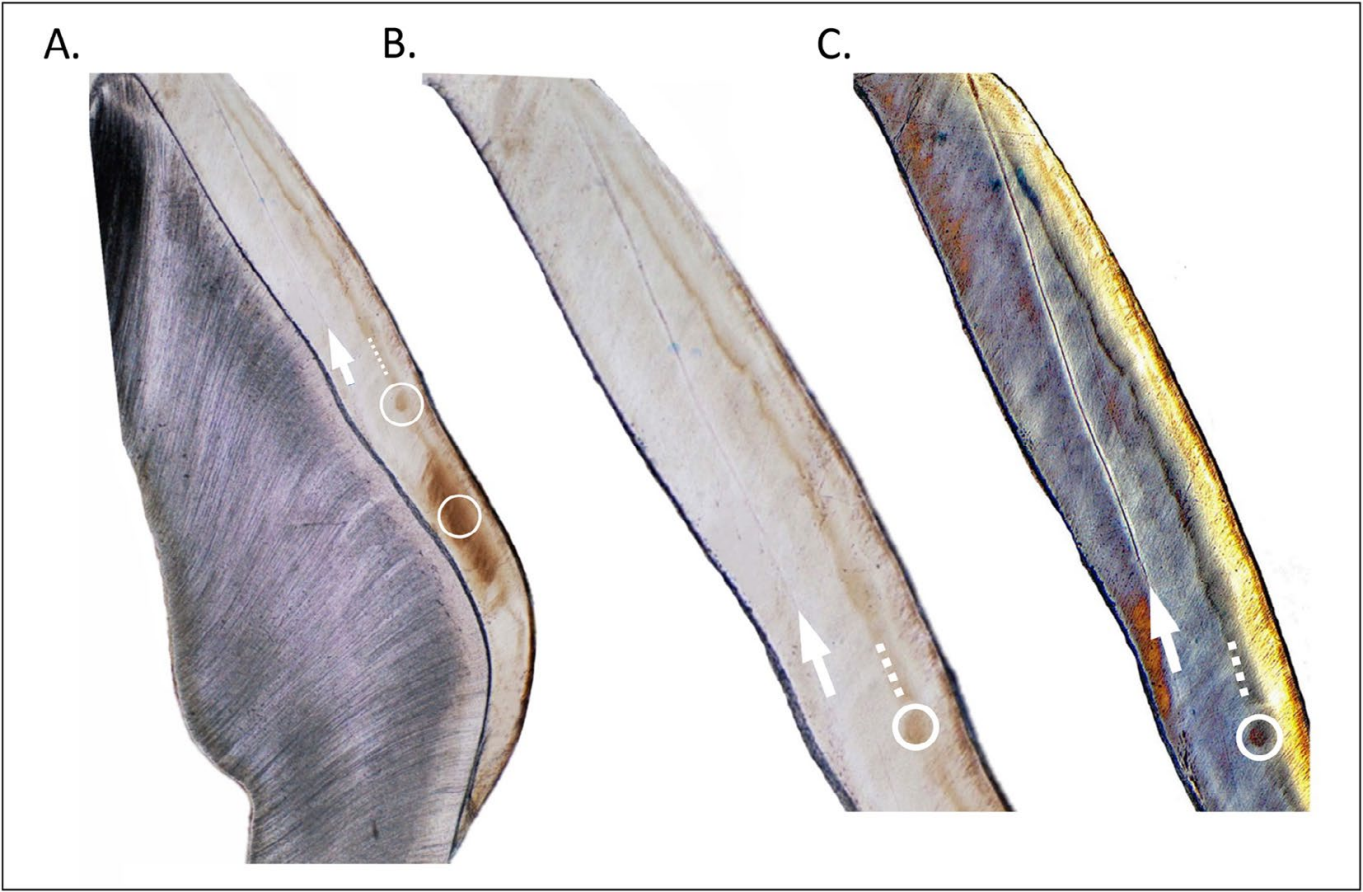
Identifying the neonatal line. Histology thin section through a Medieval deciduous molar cusp. (A) White arrow points to a faint neonatal line in enamel imaged at magnification of 4×. The two white circles indicate enamel with different mineralization properties that produced a relatively darker color. The white dashed line traces a discoloration near the outermost enamel—see text for discussion. (B) A portion of the Medieval deciduous molar enamel cusp that been enlarged showing the same faint neonatal line indicated by the white arrow. (C) The same portion of enamel re‐imaged using polarized light microscopy. The birefringent (different refractive) properties of the neonatal line (white arrow) produce a different color to the surrounding enamel making the neonatal line much more prominent. Differences in birefringence also occur between the outer enamel surface to the right of the white dashed line and the inner enamel.

We identified the NNL under the microscope using two steps. We accepted the first prominent accentuated line in cuspal (common for dm2) or lateral (dm1) enamel as the NNL. Figure 1B shows an example of a prominent NNL. Faint growth lines are present in prenatal enamel to the right of the NNL, and faint accentuated and Retzius lines are present in postnatal enamel to the left (Figure 1B). The growth lines in prenatal enamel are typical, in that when they occur, they are relatively indistinct compared to the NNL (Kurek et al. 2020; Sipovac et al. 2023). Second, in cases where the NNL was faint or diffuse, we used polarized light microscopy to produce a birefringent band that differed in color compared to the surrounding enamel. An example of a faint neonatal line that is greatly enhanced through polarized light microscopy is shown in Figure 3 (also see Mahoney 2011, his fig. 6).

Figure 3 illustrates the difference between the NNL compared to a marking that occurred after the secretory phase of enamel growth. The white dashed lines (Figure 3A–C) trace a discoloration near the outermost external enamel surface (see Martirosyan et al. 2025 for enamel diagenesis, and Weerheijm 2004 for early maturation stage developmental defects). The discoloration is not an incremental growth line nor an accentuated marking that occurred during the secretory phase of enamel growth. Secretory phase growth lines and markings follow the geometry and orientation of enamel structures, which can be seen in the shape of the neonatal line. The NNL in Figure 3, and Figure 1B, slopes downwards and inwards, from cuspal enamel at the top of the figure, until it intersects with the EDJ in lateral enamel towards the bottom of the figure. The neonatal line follows this shape because it was produced by secretory ameloblasts at the forming enamel front, during a stress event, which in this instance was birth. The discoloration indicated by the dashed white lines in Figure 3A–C does not follow the geometry of forming enamel, and instead remains generally parallel, and with a similar depth, relative to the outermost external surface.

Prenatal formation times were calculated here for *n* = 265 molars (see Results). Of these, *n* = 242 were new and recorded for this study. A total of *n* = 23 paracone prenatal formation times were reused and recalculated from Mahoney (2015) for the Medieval upper first (*n* = 10) and second molars (*n* = 13). These prenatal formation times in Mahoney (2015) were recorded using the same methodology (prism lengths and secretion rates) as the present study. For the *n* = 91 samples where we did not calculate prenatal formation times, the line either was not preserved or it was faint and other accentuated markings were present and positioned adjacent to the neonatal line undermining certainty in identification and thus were excluded. Of the *n* = 265 where the line was securely identified, identification ranged between 80% for present‐day dm2 (100 out of 125) and 78% for the archaeological dm2 (51 out of 65) compared to 66% of present‐day dm1 (60 out of 91) and 72% of archaeological dm1 (54 out of 75). Thus, the neonatal line was identified in slightly more second molars compared to first molars.

Prenatal formation time was calculated by measuring enamel thickness along an enamel prism between the tip of the dentin horn and the neonatal line (e.g., Mahoney 2011). This prism length was divided into three areas of equal thickness (inner, mid, and outer). The prism length in each area was divided by a mean DSR calculated for that area. The three formation times were summed to give prenatal formation time. Prenatal time was subtracted from enamel total Cu‐Ft to calculate post‐natal formation time for each molar.

#### 2.3.5 Scaling Lateral Enamel Formation Time

The time required to form each segment of the EDJ was calculated from a mean of the two extension rates for that segment (EDJ segment length divided by the extension rate = EDJ segment formation time in days) (Section 2.3.2). Segment formation time was then recalculated as a *proportion* of the total time taken to form the height of the EDJ. These proportions (%) were applied to the lateral enamel formation times that had been calculated previously using standard methods (Section 2.3.3 above). An example of this scaling calculation for dm2 and dm1 is given in Appendix 1.

We tested this scaling methodology on a sample of European permanent first molars (M1). Segments of M1 lateral enamel formation time were calculated using our extension rate methodology, and then compared to formation times published by others that have used either counts of Retzius lines or perikymata (Reid and Dean 2006; Guatelli‐Steinberg and Reid 2008; Modesto‐Mata et al. 2020). Our M1 extension rate data are calculated on the same thin sections utilized by Reid and Dean (2006).

#### 2.3.6 Average Enamel Thickness

A two‐dimensional measure of crown average enamel thickness (AET) was calculated from the thin sections using standard measurements (Martin 1985). The AET in mm was determined from the area of the enamel cap divided by the length of the EDJ (see Mahoney 2010: his Figure 3). This gives the average straight‐line thickness between the EDJ and the outer enamel surface in mm.

### 2.4 Statistical Analyses

Data are described through mean values, standard deviations, and ranges. Analyses were undertaken in three stages. First, significant variation amongst the present‐day samples, and amongst the archaeological samples, was assessed using a non‐parametric Kruskal–Wallis test with Dunn–Bonferroni pairwise comparisons that adjust significance values for multiple tests. Non‐parametric tests were chosen because sample sizes varied between the different tooth types and populations (see Table 1 for *n* of each population subdivided by tooth type). Second, non‐parametric Mann–Whitney *U* tests were employed to compare the grouped present‐day populations to the grouped archaeological samples. Following this, Kruskal–Wallis/Dunn–Bonferroni pairwise comparisons were used to identify which present‐day populations differed from the archaeological samples. Third, linear regression was utilized to model the relationships between total Cu‐Ft (dependent variable), and prenatal and postnatal enamel growth (independent variables) to determine if total formation time could be predicted from either of the independent variables. All statistical analyses were undertaken in IBM SPSS version 29.

## 3 Results

### 3.1 Enamel Cusp Formation Times

**FIGURE 4:**
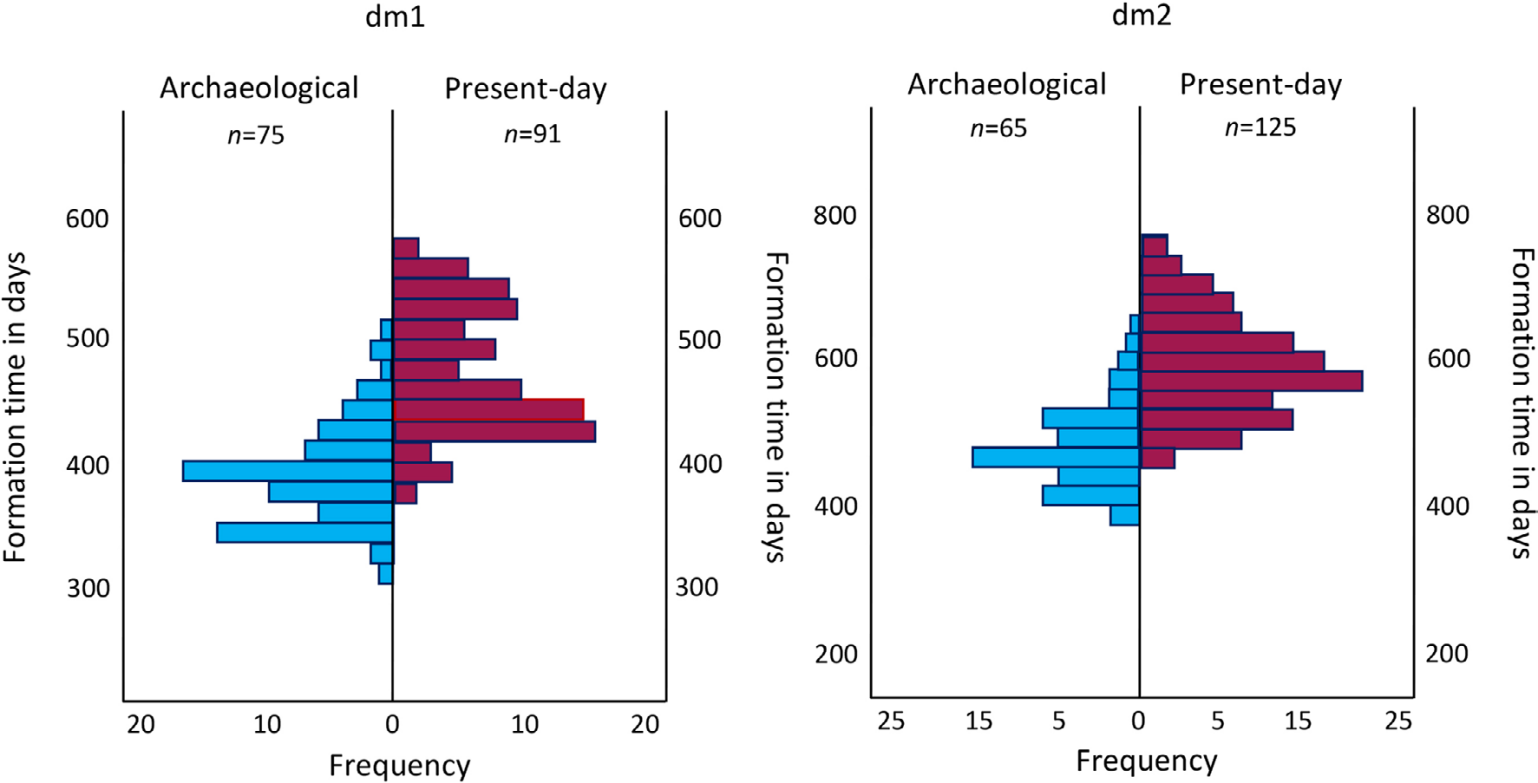
The frequency of cusp total formation times. The chart illustrates formation times for deciduous first (dm1) and second (dm2) molars from the combined archaeological periods, and the combined sample of present‐day populations.

**FIGURE 5:**
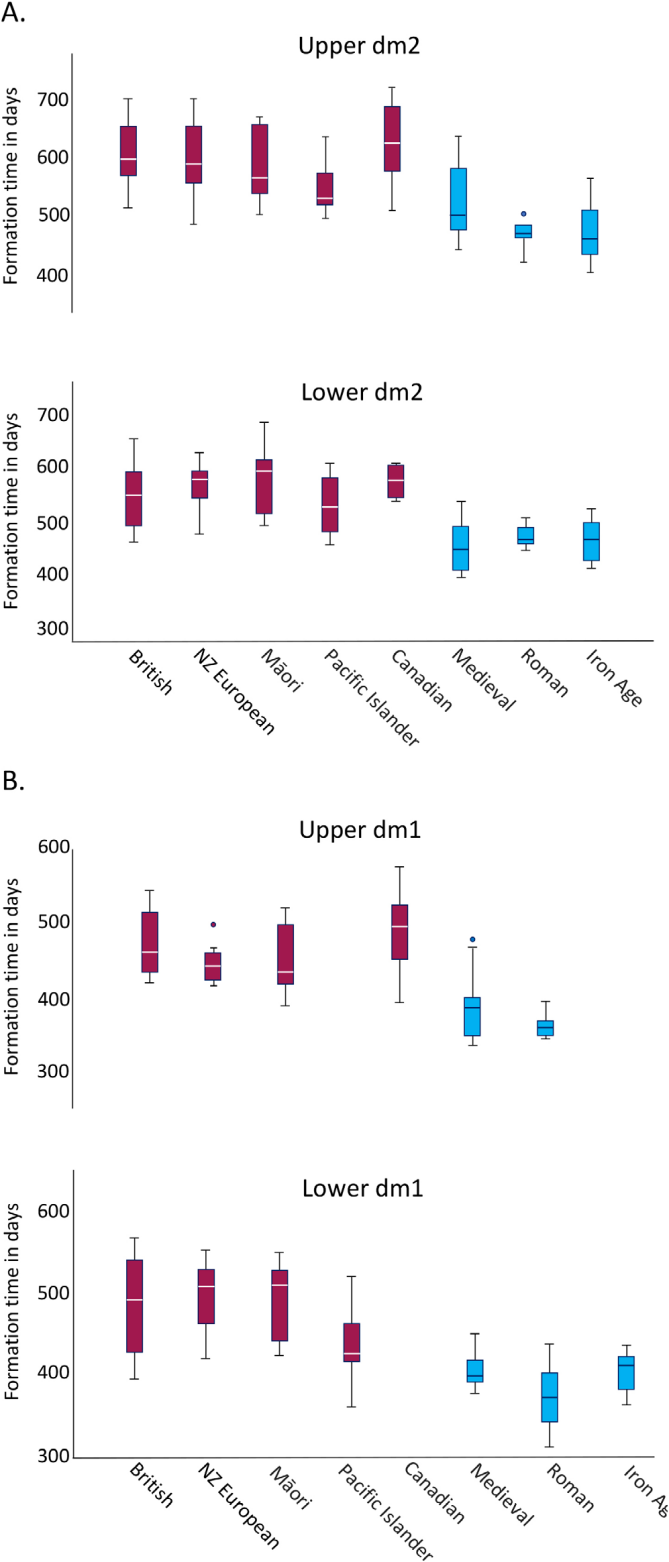
Deciduous molar enamel cusp total formation times. (A) Second molars compared between present‐day and archaeological populations. (B) First molars compared between present‐day and archaeological populations. Box plots show the median, interquartile and min‐max range.

Descriptive statistics for mean total enamel Cu‐Ft subdivided by tooth type and population are presented in Table 2 and are depicted in Figures 4 and 5. Appendix 2 shows all pairwise comparisons of Cu‐Ft and associated *p* values. Variation in total, pre‐ and postnatal enamel Cu‐Ft amongst, and between populations, is discussed below.

**TABLE 2:** Mean total cusp formation time in days (sd).

Population	Upper dm2		Lower dm2		Upper dm1		Lower dm1	
	*n*	Formation time	*n*	Formation time	*n*	Formation time	*n*	Formation time
Combined								
All	109	560 (77)	81	518 (71)	70	439 (59)	96	428 (62)
Modern								
All	74	593 (62)	51	556 (56)	48	462 (46)	43	476 (53)
British	22	605 (54)	11	545 (62)	12	470 (43)	14	482 (59)
NZ‐European	20	602 (69)	16	561 (41)	11	447 (33)	11	493 (42)
Māori	11	579 (64)	10	578 (66)	14	450 (46)	10	480 (48)
Pacific Island	11	543 (42)	8	524 (57)	—		8	435 (44)
Canada	10	619 (46)	6	567 (20)	11	482 (55)	—	—
Archaeological								
All	35	488 (57)	30	454 (45)	22	388 (50)	53	390 (36)
Medieval	14	517 (65)^1^	18	447 (46)	17	398 (55)^1^	15	408 (24)
Roman	5	473 (20)	6	474 (16)	5	354 (12)	28	373 (35)
Iron Age	16	464 (45)	6	437 (27)	—	—	10	413 (34)

*Note:* 1 = Recalculated from Mahoney (2015).

#### 3.1.1 Second Molar Enamel Total cu‐Ft

##### 3.1.1.1 Modern Humans

The mean total enamel Cu‐Ft for the entire sample of *n* = 190 human deciduous second molars (all present‐day and archaeological combined) was 542 days (sd = 77) with a range that extended from a lowermost value of 388 days to an uppermost value of 701 days (dm^2^ mean = 560 days, range = 398–701 days; dm_2_ mean = 518, range = 388–680 days; Table 2).

##### 3.1.1.2 Amongst present‐day and archaeological samples

No significant differences were found in lower dm2 total Cu‐Ft when compared amongst the present‐day samples, or amongst the archaeological samples (Appendix 2). Upper dm2 Cu‐Ft varied significantly amongst present‐day populations (*X* ^2^(4) = 11.006, *p* = 0.027), but not within the archaeological periods. Pairwise comparisons revealed upper dm2 from Pacific Island peoples formed over a significantly shorter period compared to British (*U* = 22.045; *p* = 0.049) and Canadian samples (*U* = 26.059, *p* = 0.048).

##### 3.1.1.3 Between present‐day and archaeological samples

Thirty‐four percent of the dm2 total Cu‐Ft from the archaeological periods extended below the lowermost value (the shortest formation time) from the present‐day populations (Figure 4). When subdivided by tooth type, 56% of upper dm2 total Cu‐Ft from the archaeological periods extended below the lowermost Cu‐Ft from the present‐day, and the difference between their mean formation times was significant (*U* = 258.500; *p* < 0.001; Table 2). Forty‐seven percent of lower dm2 Cu‐Ft from the archaeological periods extended below those from the present‐day and the difference between the mean values was significant (*U* = 109.500; *p* < 0.001; Table 2).

Pairwise comparisons revealed Roman and Iron Age upper dm2 had a significantly lower total Cu‐Ft compared to NZ‐Europeans, British and Canadian samples (Figure 5A; Appendix 1 for *p* values). Iron Age samples had a significantly lower Cu‐Ft than Māori samples. The Medieval upper dm2 sample had a significantly lower formation time compared to British and Canadian samples. Medieval lower dm2 total Cu‐FT was significantly less than all present‐day populations except Pacific Island peoples (Figure 5A; Appendix 2). The Iron Age lower dm2 was significantly lower than the NZ‐European and Māori samples. The Roman Cu‐Ft was significantly lower than the Canadian sample, and the adjusted *p* values approached significance compared to NZ Europeans (*p* = 0.093) and Māori (*p* = 0.072).

#### 3.1.2 First Molar Enamel Total cu‐Ft

##### 3.1.2.1 Modern Humans

When the entire sample of *n* = 166 upper and lower first molars from all populations was combined, the mean total Cu‐Ft was 432 days (sd = 61) with a range between 313 days and 573 days (dm^1^ mea*n* = 439 days, range = 336–573 days; dm_1_ mean = 428, range = 313–570 days; Table 2).

##### 3.1.2.2 Amongst Present‐Day and Archaeological Samples

No significant differences in total Cu‐Ft were found when comparisons were undertaken within present‐day populations for either upper or lower dm1 (Appendix 2). Lower dm1 Cu‐Ft differed significantly within the archaeological samples (*X* ^2^(2) = 11.928, *p* = 0.003). Pairwise comparisons revealed the Roman dm_1_ total Cu‐Ft was significantly lower compared to the Medieval (*p* = 0.010) and Iron age periods (*p* = 0.029). Upper dm1 did not vary significantly within the archaeological periods.

##### 3.1.2.3 Between Present‐Day and Archaeological Samples

Twenty‐five percent of the dm1 total Cu‐Ft values from the archaeological periods were lower than the lowermost value from present‐day populations (Figure 4). When subdivided by tooth type, 59% of the total Cu‐Ft for upper first molars from the archaeological periods extended below those from present‐day populations and the difference between their mean formation times was significant (*U* = 146.000; *p* < 0.001). Twenty percent of the total Cu‐FT from the archaeological lower dm1 extended below those from present‐day populations and the difference between mean values was significant (*U* = 202.500; *p* < 0.001).

Pairwise comparisons revealed the total Cu‐Ft of Roman upper dm1 samples was significantly lower compared to each of the present‐day samples (Figure 5B; Appendix 2). Medieval upper dm1 differed significantly compared to British and Canadian populations but not the Māori or NZ‐Europeans. Pairwise comparisons revealed the total Cu‐Ft mean of Roman lower dm1 was significantly less compared to British, Māori and NZ‐European samples (Figure 5B; Appendix 2). Medieval lower dm1 was significantly lower compared to the NZ‐Europeans only, and the adjusted *p* values approached significance when compared to British (*p* = 0.071) and Māori (*p* = 0.079) samples. Iron Age lower dm1 did not differ significantly compared to the present‐day populations.

#### 3.1.3 Second Molar Enamel Postnatal cu‐Ft

**FIGURE 6:**
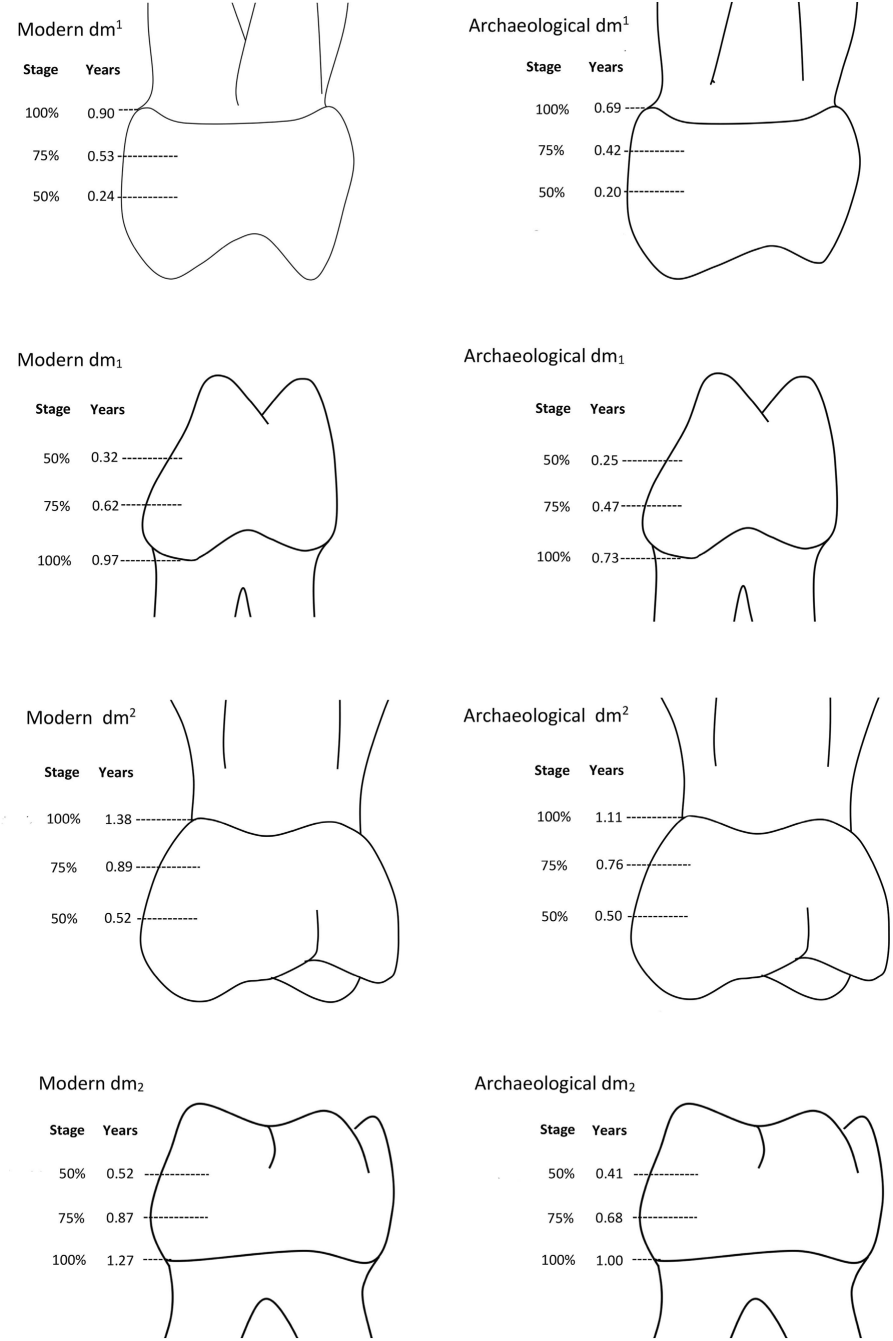
Mean postnatal enamel cusp formation times. The combined present‐day (modern) populations are to the left and combined archaeological samples are to the right. Stages of cusp completion are 50%, 75%, and cusp complete indicated by 100%. Formation times are mean values for each stage and are given in years after birth. Data taken from Tables 3 and 4. Ranges for each completion stage can be calculated from standard deviations in these tables.

**FIGURE 7:**
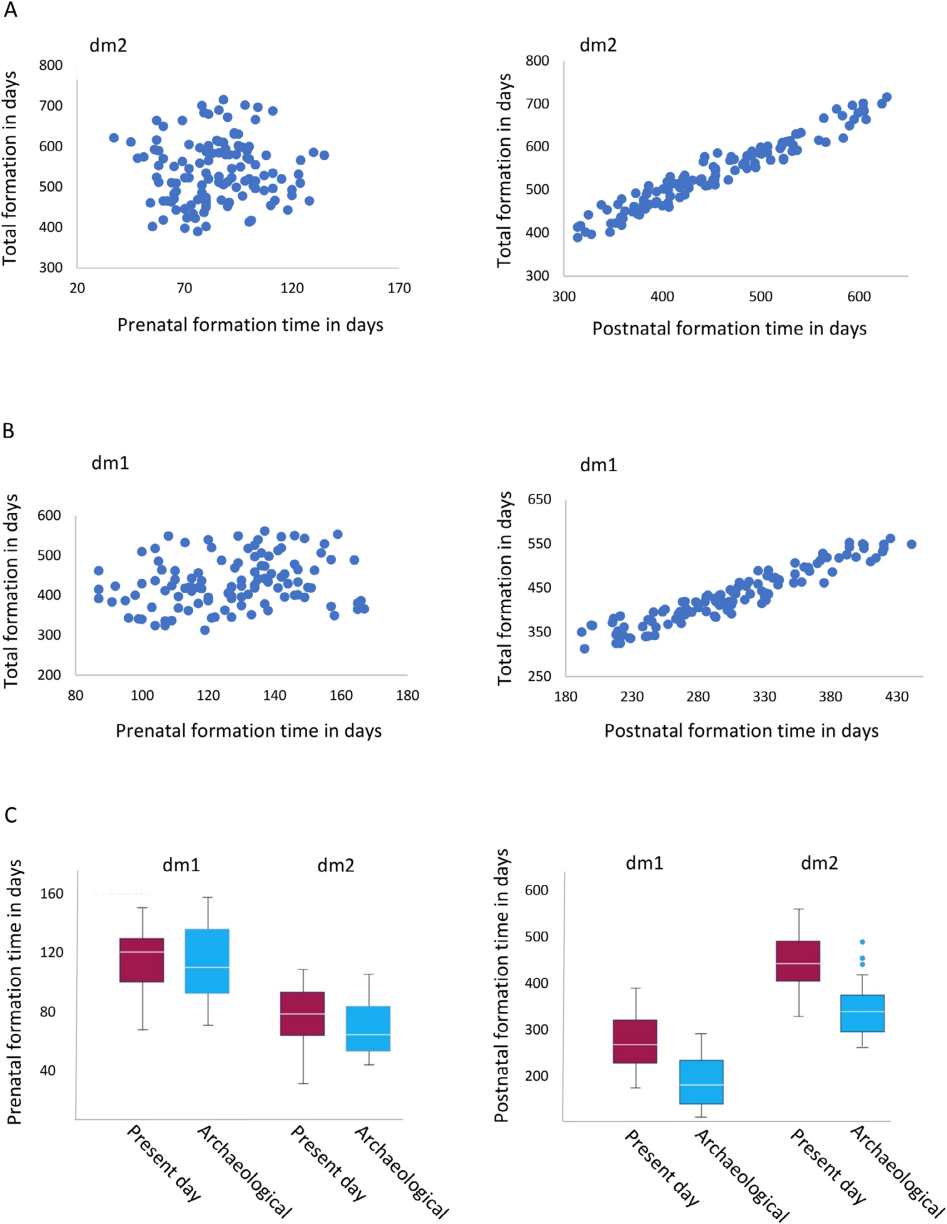
Prenatal and postnatal enamel formation time related to total formation time. Prenatal is to the left and postnatal is to the right of each row. See text for statistics. (A) The total Cu‐Ft of dm2 was not related to prenatal formation time but it was significantly related to the postnatal formation time of this tooth type. (B) The total Cu‐Ft of dm1 was not related to prenatal formation time but it was significantly related to postnatal formation time. (C) Prenatal formation times did not differ between the present day and archaeological samples, but postnatal formation times of dm1 and dm2 differed significantly between these samples.

The neonatal line was securely identified in 265 molars out of the entire sample of 356 . Prenatal and postnatal formation times were calculated for these 265 molars (dm1 = 114: present‐day = 52, archaeological = 62; dm2 = 151: present day = 100, archaeological = 51). Descriptive statistics for all postnatal formation times are given in Tables 3 and 4 and are illustrated in Figures 6 and 7. Inferential statistics and *p* values for comparisons between populations subdivided by tooth type are shown in Appendix 3.

**TABLE 3:** Prenatal and postnatal enamel formation time in days (sd) for present‐day deciduous molars.

	All	British	NZ Europe	Māori	Pacific Isles	Canadian
UPPER dm2						
Before birth						
Initiation	92 (16)	103 (16)	88 (15)	84 (13)	94 (16)	87 (20)
After birth						
Cuspal complete	45 (41)	21 (42)	47 (43)	45 (53)	34 (33)	69 (39)
50% crown length	188 (40)	172 (38)	190 (50)	181 (53)	169 (36)	214 (41)
75% crown length	323 (50)	315 (45)	328 (57)	310 (48)	292 (35)	350 (44)
100% crown length	501 (62)	502 (54)	514 (69)	494 (64)	449 (42)	532 (46)
LOWER dm2						
Before birth						
Initiation	91 (19)	91 (18)	95 (23)	90 (12)	88 (10)	76 (12)
After birth						
Cuspal complete	47 (32)	47 (25)	45 (36)	74 (25)	38 (24)	61 (38)
50% crown length	192 (37)	194 (30)	195 (34)	222 (38)	175 (33)	210 (29)
75% crown length	318 (40)	312 (43)	325 (40)	351 (50)	296 (37)	340 (20)
100% crown length	464 (56)	454 (62)	477 (41)	500 (66)	436 (57)	491 (20)
UPPER dm1						
Before birth						
Initiation	134 (15)	143 (11)	138 (9)	124 (13)	—	134 (21)
Cuspal complete	31 (30)	47 (20)	18 (26)	21 (35)		6 (25)
After birth						
50% crown length	87 (30)	77 (25)	90 (31)	94 (29)	—	112 (27)
75% crown length	192 (29)	186 (28)	185 (25)	195 (30)	—	215 (34)
100% crown length	328 (46)	327 (43)	309 (33)	326 (46)	—	348 (55)
LOWER dm1						
Before birth						
Initiation	124 (20)	118 (16)	133 (18)	121 (23)	125 (13)	—
Cuspal complete	16 (21)	13 (20)	17 (18)	12 (24)	10 (12)	—
After birth						
50% crown length	113 (26)	119 (33)	123 (21)	126 (26)	102 (19)	—
75% crown length	224 (30)	233 (27)	228 (29)	234 (21)	198 (30)	—
100% crown length	352 (53)	364 (59)	360 (42)	361 (48)	310 (44)	—

**TABLE 4:** Prenatal and postnatal enamel formation time in days (sd) for archaeological deciduous molars.

	All	Medieval	Roman	Iron Age
UPPER dm2				
Before birth				
Initiation	80 (20)	81 (21)^1^	90 (21)	72 (10)
After birth				
Cuspal complete	84 (50)	81 (41)	61 (23)	89 (51)
50% crown length	186 (36)	197 (38)	161 (19)	175 (40)
75% crown length	278 (43)	297 (51)	252 (19)	264 (34)
100% crown length	408 (57)	439 (65)	383 (20)	392 (45)
LOWER dm2				
Before birth				
Initiation	85 (21)	86 (20)	83 (23)	84 (21)
After birth				
Cuspal complete	37 (35)	35 (24)	48 (35)	64 (33)
50% crown length	148 (33)	143 (27)	159 (18)	160 (21)
75% crown length	248 (20)	242 (30)	260 (18)	249 (27)
100% crown length	370 (45)	361 (46)	391 (12)	354 (27)
UPPER dm1				
Before birth				
Initiation	135 (24)	138 (25)^1^	130 (13)	—
Cuspal complete	18 (34)	23 (34)	14 (13)	—
After birth				
50% crown length	74 (28)	89 (30)	74 (9)	—
75% crown length	152 (40)	164 (46)	140 (11)	—
100% crown length	252 (54)	260 (55)	224 (13)	
LOWER dm1				
Before birth				
Initiation	123 (19)	130 (15)	119 (17)	123 (27)
Cuspal complete	6 (20)	8 (17)		11 (25)
After birth				
Cuspal complete			6 (22)	
50% crown length	92 (20)	69 (12)	94 (18)	97 (21)
75% crown length	173 (25)	162 (20)	167 (26)	185 (29)
100% crown length	267 (37)	280 (29)	254 (34)	290 (34)

##### 3.1.3.1 Modern Humans

When the entire sample of second molars was combined, the mean post‐natal Cu‐Ft was 452 days (sd = 80 days) after birth with a range between a minimum value of 314 days and a maximum of 628 days (dm^2^ mean = 467 days, range = 328 to 628 days; dm_2_ mean = 435, range = 314 to 598 days; Tables 3 and 4). On average, 84% of dm2 enamel formed after birth.

##### 3.1.3.2 Amongst Present‐Day and Archaeological Samples

Postnatal upper and lower dm2 formation times did not differ significantly when compared amongst the present‐day samples, or amongst the archaeological periods (Appendix 3). Linear regression analysis revealed that second molar postnatal formation time was significantly and positively related to total Cu‐Ft (*r* ^2^ = 0.938; *p* < 0.001; Figure 7a). The regression equation for predicting dm2 total Cu‐Ft from postnatal formation times is:

- dm2 total Cu‐Ft in days = 108.02 + (0.949 × postnatal formation time).

##### 3.1.3.3 Between Present‐Day and Archaeological Samples

Twenty‐nine percent of upper dm2 post‐natal Cu‐Ft from the combined archaeological samples extended below the lowermost value from present‐day populations. Upper dm2 from the combined archaeological periods was complete on average 13.4 months after birth which was significantly earlier compared to upper dm2 from the combined present‐day populations which completed formation 16.5 months after birth (U = 133.500; *p* = 0.001) (Tables 3 and 4; Figures 6 and 7C).

Pairwise comparisons reveal upper dm2 from the Roman and Iron Age period had a significantly shorter postnatal formation time compared to the NZ‐Europeans and Māori samples (Appendix 3). The Iron Age upper dm2 samples also had a significantly shorter formation time compared to Canadian samples. Medieval upper dm2 postnatal formation time was significantly shorter compared to Canadian samples and the adjusted *p* value approached significance (*p* = 0.064) when compared to NZ‐Europeans.

Thirty‐eight percent of the lower dm2 postnatal Cu‐Ft from the combined archaeological samples lies below the lowermost range of present‐day samples. Lower dm2 from the combined archaeological periods completed formation significantly earlier, 12.1 months after birth compared to present‐day samples that required an average of 15.3 months after birth to complete formation (*U* = 69.000; *p* < 0.001) (Tables 3 and 4; Figures 6 and 7C). Pairwise comparisons revealed lower dm2 postnatal formation of the Medieval period was significantly lower compared to the NZ‐Europeans. The Medieval, Iron Age and Roman samples had a significantly shorter postnatal formation period compared to Māori.

#### 3.1.4 First Molar Enamel Postnatal Cu‐Ft

##### 3.1.4.1 Modern Humans

When all first molars (upper and lower) from all populations were combined, the mean post‐natal Cu‐Ft was 305 days (sd = 60 days) after birth with a range between a minimum value of 192 days and a maximum value of 441 days (dm^1^ mean = 306 days, range = 192 to 420 days; dm_1_ mean = 304, range = 194 to 441 days; Tables 3 and 4). On average, 71% of dm1 enamel formed after birth.

##### 3.1.4.2 Amongst Present‐Day and Archaeological Samples

Postnatal formation times did not differ significantly amongst the present‐day samples, or amongst the archaeological periods, though the lower dm1 from Iron Age samples approached significance (*p* = 0.062) when compared to the Roman period (Appendix 3). Linear regression analysis revealed first molar postnatal formation time was significantly and positively related to total Cu‐Ft (*r* ^2^ = 0.901; *p* = 0.001; Figure 7B). The regression equation for predicting dm1 total Cu‐Ft from postnatal formation time is:

- dm1 total Cu‐Ft in days = 136.08 + (0.971 × postnatal formation time).

##### 3.1.4.3 Between Present‐Day and Archaeological Samples

Sixty percent of all upper dm1 post‐natal Cu‐Fts from the combined archaeological samples extended below the lowermost value for present‐day populations. Upper dm1 from the combined archaeological populations was completed significantly earlier, 8.4 months after birth, compared to present‐day samples that completed formation 10.8 months after birth (*U* = 78.000; *p* = 0.001; Figures 6 and 7C). Pairwise comparisons reveal upper dm1 from the Medieval and Roman period, but not the Iron Age, had a significantly shorter post‐natal formation time compared to NZ‐Europeans, Māori, and British samples but not the Pacific Island peoples (Appendix 2).

Thirty‐nine percent of the post‐natal Cu‐Fts for lower dm1 from the combined archaeological periods lay below the lowermost range of present‐day samples. Lower dm1 from the archaeological periods completed formation significantly earlier, 8.8 months after birth, compared to present‐day samples that formed over an average of 11.6 months (*U* = 98.500; *p* < 0.001) (Tables 3 and 4; Figure 6). Pairwise comparisons reveal lower dm1 from Roman samples was significantly less than Canadian and British samples.

#### 3.1.5 Prenatal Enamel Formation Time

Descriptive statistics for each population subdivided by tooth type are presented in Tables 3 and 4 and are illustrated in Figure 7. Appendix 4 shows pairwise comparisons and associated *p* values.

##### 3.1.5.1 Modern Humans

When all dm2 (upper and lower) from all populations were combined, prenatal formation ranged between 37 and 135 days with an average initiation age of 88 days before birth. When all dm1 were combined, prenatal formation times ranged between 87 and 167 days with an average initiation age of 127 days before birth.

##### 3.1.5.2 Amongst Present‐Day and Archaeological Samples

Mean prenatal formation time did not differ significantly within present‐day populations, or within the archaeological period. Regression analyses revealed prenatal formation time was not significantly related to total Cu‐Ft for dm2 (*r* ^2^ = 0.002; *p* < 0.619; Figure 7A) or dm1 (*r* ^2^ = 0.042; *p* = 0.056; Figure 7B).

##### 3.1.5.3 Between Present‐Day and Archaeological Samples

Lower dm2, and both upper and lower first molar prenatal formation times did not differ significantly between present‐day populations compared to any archaeological period. Prenatal formation time of British upper second molars was significantly greater compared to Iron Age samples (*p* = 0.002).

### 3.2 Growth Rates

#### 3.2.1 Enamel Secretion Rates

**FIGURE 8:**
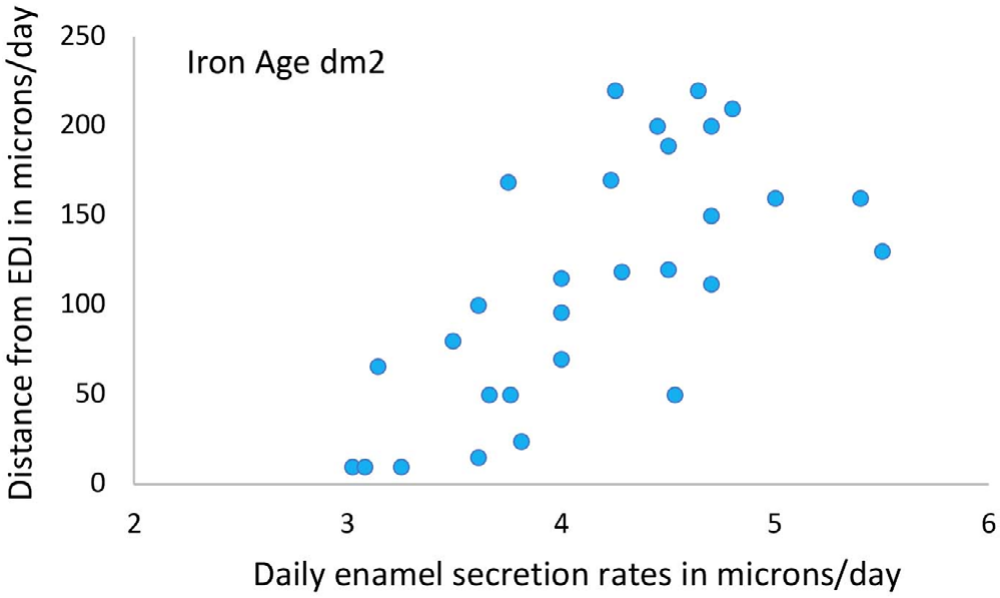
Daily enamel secretion rates. Rates are given in μm/day for the first formed 250 μm of prenatal cuspal enamel for the Iron Age sample.

When cuspal enamel was subdivided into three regions of equal thickness (inner, mid, outer), mean DSRs increased from inner to mid enamel regions for all archaeological samples (Table 5). Rates either increased or decreased slightly into the outermost enamel compared to the mid enamel region. Our more detailed analysis of enamel formation for the Iron Age dm2 samples (first formed enamel over approximately 60 days) revealed secretion rates were lowest when adjacent to the EDJ (within 25 μm) where DSRs ranged between 2.90 and 3.25 μm/day. Rates then accelerated quickly and were typically around 4 μm/day after 100 μm of enamel had formed (Figure 8). Thus, the slower rates near the EDJ contributed to the slower overall mean rate we report for the inner one third of enamel thickness.

**TABLE 5:** Mean daily secretion rates in μm/day for regions of cuspal enamel.

		*n*	Inner	Mid	Outer
dm1	Medieval	12–33	3.80 (0.36)	4.40 (0.42)	4.49 (0.50)
	Roman	20–28	4.23 (0.38)	4.56 (0.41)	4.73 (0.51)
	Iron age	10	4.15 (0.54)	4.39 (0.49)	5.30 (0.45)
dm2	Medieval	10–21	3.98 (0.35)	4.53 (0.50)	5.15 (0.49)
	Roman	8–11	4.18 (0.55)	4.41 (0.44)	4.22 (0.48)
	Iron age	15	4.07 (0.52)	4.58 (0.40)	4.47 (0.61)

#### 3.2.2 Enamel Extension Rates

**FIGURE 9:**
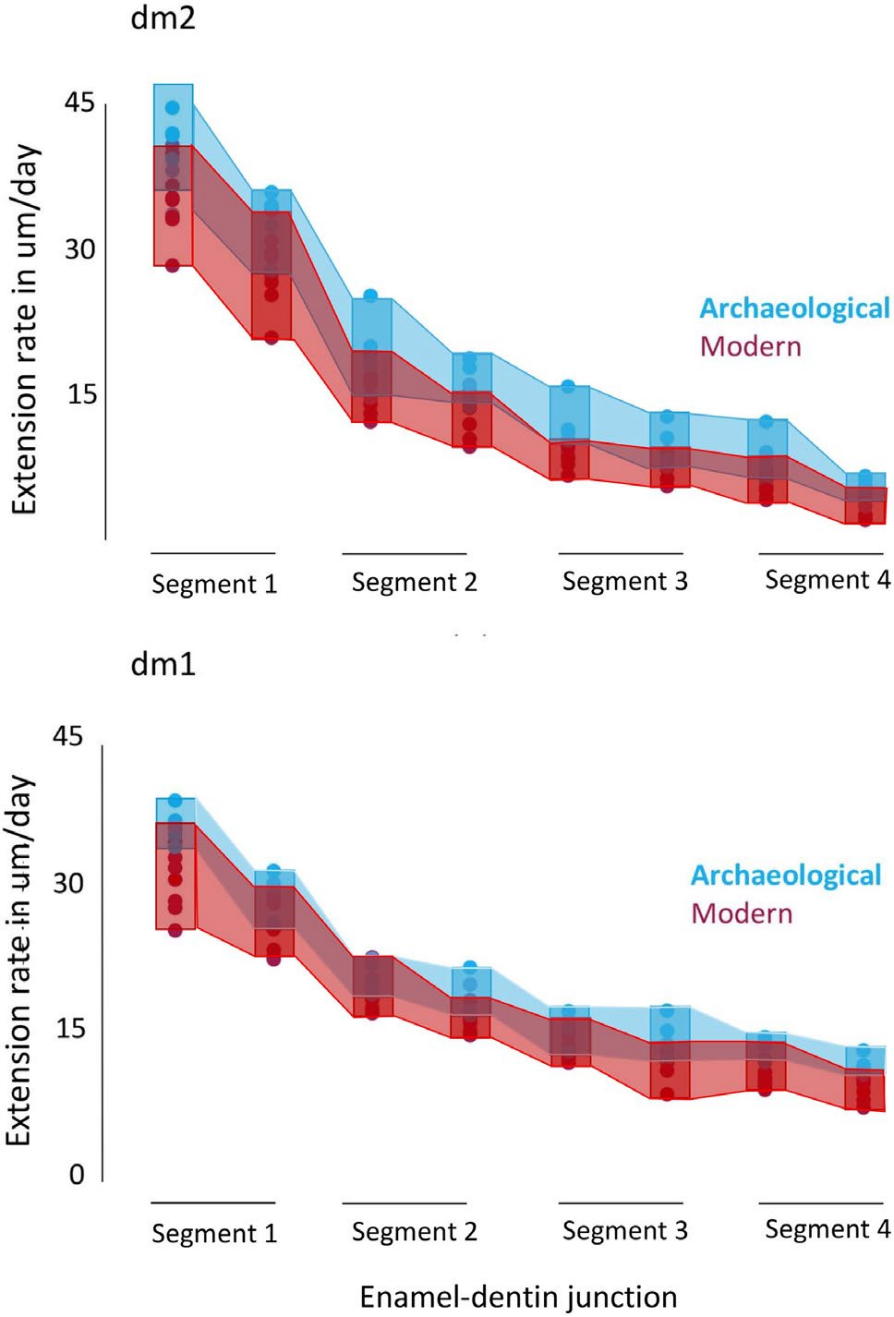
Mean enamel extension rates. Rates are given in μm/day for four equal segments of the EDJ. Segment 1 is nearest the dentin horn and segment 4 is nearest the cervix. See text for the methodology and Appendixes 5 and 6 for data.

Figure 9 illustrates the mean extension rates within each of the four equal segments of the EDJ for dm1, and dm2. Table 6 shows a mean of the two values for each molar type for each segment of the EDJ. Appendixes 5 and 6 give the extension rate data and standard deviations for each tooth type for each population.

**TABLE 6:** Mean extension rate in μm/day for each segment of the EDJ.

Segment	Present day		Archaeological	
	Upper dm1	Lower dm1	Upper dm1	Lower dm1
1	28.95	26.44	33.64	32.59
2	17.84	17.76	19.81	19.73
3	12.98	12.34	14.59	14.77
4	9.43	10.64	11.50	12.71

Segment	Present day		Archaeological	
	Upper dm2	Lower dm2	Upper dm2	Lower dm2
1	33.10	29.76	37.18	34.38
2	17.44	15.63	20.40	19.45
3	12.14	11.78	14.50	13.59
4	9.20	10.18	10.10	11.37

As expected, growth in height commenced rapidly and then slowed towards the cervical enamel region in segment four for all samples (Figure 9). The mean initial rate in Segment 1, nearest the dentin horn (first of two), was faster in upper molars compared to lower molars. When subdivided and compared amongst present‐day populations, Pacific Island peoples had the highest initial segment one extension rate, but differences were inconsistent when rates were compared between other segments.

The initial rate in Segment 1 for archaeological samples ranged between 35.16 and 39.53 μm/day in upper dm1 and 34.68 and 36.93 μm/day in lower dm1, which extended above the mean initial rates of the British, NZ Europeans and Māori samples which lay between 28.99 and 33.56 μm/day (Appendix 5). Mean initial rates for upper dm2 of archaeological samples (37.55 to 42.84 μm/day) and lower dm2 (37.38 to 40.46 μm/day), extended above those from British, NZ Europeans, and Māori samples (upper dm2 = 33.17 to 36.11 μm/day; lower dm2 = 29.11 to 33.57 μm/day; Appendix 6). Initial rates for Pacific Island peoples lower dm1 (37.40 μm/day) and upper and lower dm2 (34.86 and 38.89 μm/day) were similar to those from the archaeological periods for these tooth types.

### 3.3 Enamel Thickness

Mean AET values for upper and lower dm1 and dm2 lie within previously published values (mean and one standard deviation) reported for deciduous molars by Mahoney (2013) for a mixed archaeological sample from the United Kingdom, and by Ortiz et al. (2020) for present‐day European and African samples.

#### 3.3.1 Modern Humans

When second molars from all populations were combined, the mean AET was 0.65 mm (sd = 0.11) (dm^2^ mean = 0.70 mm, sd = 0.11; dm_2_ mean = 0.60 mm, sd = 0.07; Table 7). When first molars from all populations were combined, the mean AET was 0.48 mm (sd = 0.06) (dm^1^ mean = 0.51 mm, sd = 0.06; dm_1_ mean = 0.46 mm, sd = 0.06).

**TABLE 7:** Average enamel thickness in mm (sd).

Population	Upper dm2		Lower dm2		Upper dm1		Lower dm1	
	*n*	AET	*n*	AET	*n*	AET	*n*	AET
Combined								
All	103	0.70 (0.11)	78	0.60 (0.06)	68	0.50 (0.06)	89	0.45 (0.07)
Modern								
All	68	0.72 (0.10)	48	0.61 (0.07)	46	0.51 (0.08)	39	0.46 (0.07)
British	21	0.73 (0.08)	11	0.61 (0.05)	12	0.51 (0.05)	14	0.46 (0.08)
NZ‐European	20	0.72 (0.11)	16	0.60 (0.07)	11	0.53 (0.06)	11	0.45 (0.06)
Māori	10	0.68 (0.08)	8	0.60 (0.06)	12	0.52 (0.07)	7	0.43 (0.04)
Pacific Island	9	0.71 (0.09)	7	0.58 (0.04)	—		7	0.46 (0.04)
Canada	8	0.72 (0.10)	6	0.62 (0.06)	11	0.48 (0.07)	—	
Archaeological								
All	35	0.67 (0.10)	30	0.58 (0.06)	22	0.50 (0.06)	50	0.45 (0.06)
Medieval	14	0.67 (0.05)	18	0.57 (0.07)	17	0.50 (0.06)	15	0.44 (0.05)
Roman	5	0.66 (0.11)	6	0.61 (0.03)	5	0.50 (0.07)	25	0.46 (0.04)
Iron Age	16	0.68 (0.08)	6	0.56 (0.06)	—	—	10	0.48 (0.07)

#### 3.3.2 Amongst Present‐Day and Archaeological Samples

**FIGURE 10:**
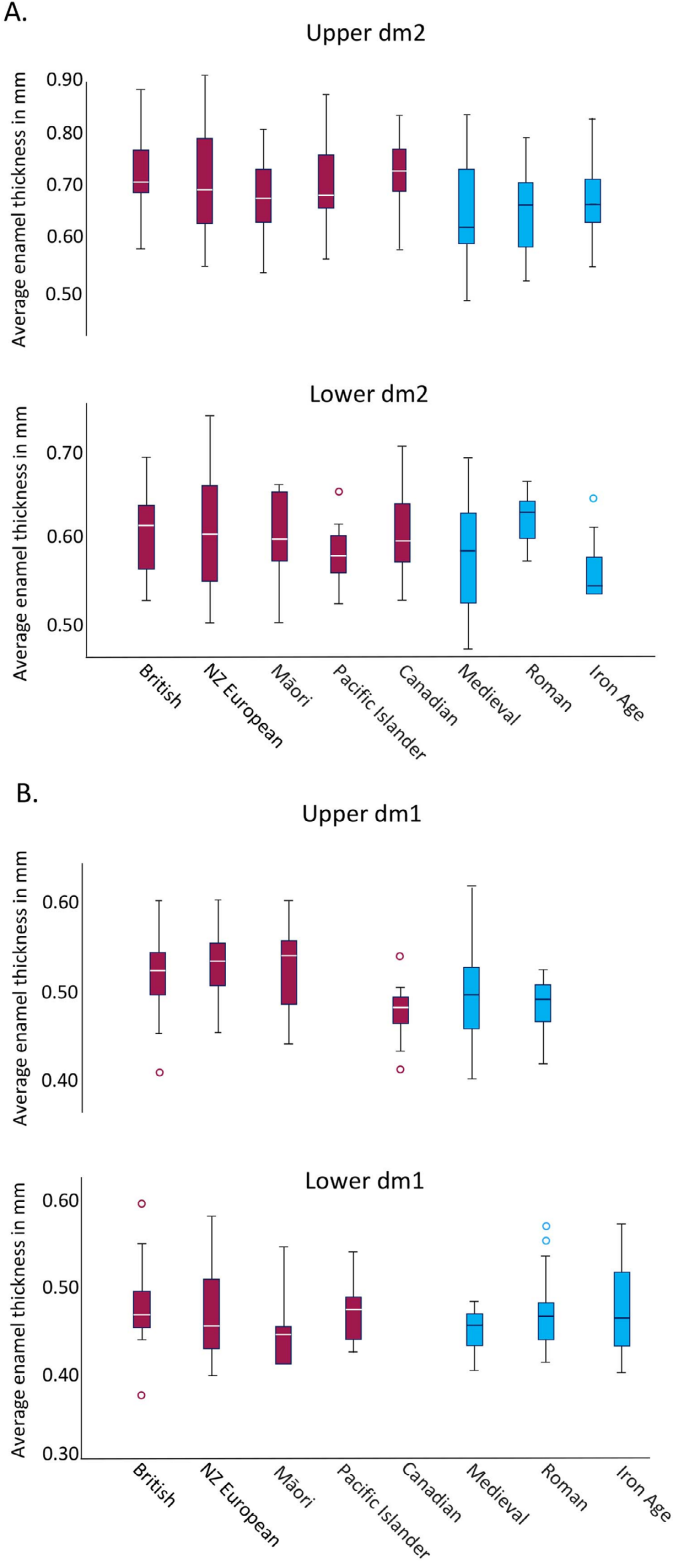
Average enamel thickness of deciduous molars. (A) Upper and lower dm2. (B) Upper and lower dm1.

No significant differences were found in either upper or lower dm2 or dm1 AET when compared amongst present‐day samples, or amongst archaeological samples (Figure 10A,B; Appendix 7 for pairwise comparisons).

#### 3.3.3 Between Present‐Day and Archaeological Samples

When the archaeological periods were combined, 8% of dm2 and 1% of dm1 AET values extended below the lowermost AET values of these tooth types from present‐day populations. There was a trend towards a slightly lower mean AET for upper dm2 from the archaeological samples compared to the present day, but the difference was not significant. Lower dm2 and both upper and lower dm1 AET did not differ significantly when compared between present‐day and archaeological samples.

### 3.4 Lateral Enamel Scaling Proportions

The mean percentage of time required to form each segment of EDJ calculated from extension rates varied only slightly when compared between the samples (Table 8). As expected, the percentage of time required to form the EDJ increased from segment one (nearest the cusp tip) to segment four (nearest the cervix). The percentages in Table 8 can be used to scale deciduous molar lateral enamel formation time (calculated from prism lengths and DSRs) into three segments (50%, 75% and 100% complete).

**TABLE 8:** Mean proportion (%) of EDJ segment formation time.

Segment	dm^1^ Modern (*n* = 32)	dm^1^ Archaeological (*n* = 20)	dm^2^ Modern (*n* = 43)	dm^2^ Archaeological (*n* = 18)
1	12.62	12.69	10.87	11.02
2	20.48	21.42	20.42	20.09
3	29.11	28.99	29.59	28.49
4	37.79	36.90	39.12	40.40

Segment	dm_1_ Modern (*n* = 35)	dm_1_ Archaeological (*n* = 35)	dm_2_ Modern (*n* = 49)	dm_2_ Archaeological (*n* = 18)
1	14.05	13.42	11.96	12.02
2	20.92	22.25	22.78	21.24
3	30.11	29.78	30.24	30.40
4	34.92	34.55	35.02	36.34

#### 3.4.1 Testing Lateral Enamel Scaling Proportions

We tested our methodology using permanent first molars. Segment formation times calculated from our extension rate methodology were most similar to formation times calculated from counts of Retzius lines or perikymata for segments two (50%), three (75%) and four (100%) (results are shown in Tables S1–S3 in Appendix 8). We utilized our extension rate methodology on the deciduous molars (in Tables 3 and 4, and Figure 6) to adjust the lateral enamel formation time calculated from standard methods using prism lengths and DSRs. Differences in segment one formation times between the different methodologies are discussed in Section 4.3 below.

## 4 Discussion

Histological studies of human enamel cusp formation time have tended to focus on permanent teeth. Here we reconstructed cusp formation times for deciduous molars from present‐day populations of different ethnicities from different geographic regions, and compared these to formation times calculated for three archaeological populations of different periods. Results reveal few differences in formation times among present‐day populations except for Pacific Island peoples. Enamel cusps of the archaeological periods formed over a shorter period after birth with relatively accelerated enamel growth rates, compared to present‐day populations. Mean prenatal formation time and average enamel thickness varied only slightly and inconsistently when compared between all populations.

### 4.1 Variation in Enamel Formation Time

#### 4.1.1 Amongst Present‐Day and Archaeological Samples

The prediction that the total formation time of Māori deciduous molars would be less than those from NZ‐Europeans was not supported by our data. The mean and range of total Cu‐Fts were remarkably similar between Canadian, British, NZ‐European and Māori populations. Our finding is equivalent to that reported for permanent molars by Reid and Dean 2006. They observed few differences in enamel completion times of permanent molar cusps from Northern Europe, North America, and South Africa (Reid and Dean 2006).

The deciduous molars of the Pacific Island peoples were the exception amongst our present‐day populations. Based upon their advanced permanent molar mineralization (Te Moananui et al. 2008), we predicted they would have shortened total formation times. We observed their upper deciduous molar cusps formed over significantly shorter periods compared to British and Canadian samples. The difference in Cu‐Ft was not significant when the NZ‐Europeans were considered, but Pacific Island peoples had a consistently lowered mean total and postnatal formation time when compared to any present‐day population (Figure 5). Their shortened postnatal formation times did not relate to a relatively longer enamel growth period before birth.

When just the archaeological samples were considered, enamel completion times varied only slightly from one period to the next, and there was no temporal trend apparent in their Cu‐Fts. The lower dm1 was the only tooth type to differ significantly, with the total Cu‐Ft for the Roman period falling below that of the Medieval and Iron Age samples. Previously reported histologically derived formation times for archaeological samples of deciduous molars are similar to those reported here (Mahoney 2011; Magri et al. 2024). The range reported by Magri et al. (2024) of 378 to 418 days for dm_1_ (*n* = 13; wear stage 1 and 2) from Medieval Italy, falls within the range of values we calculated (370 to 453 days) for lower dm_1_s (*n* = 17) from Medieval England. Our range of Cu‐Fts between 388 and 432 days for Medieval lower dm_1_ overlaps with the range of 373 to 403 days reported by Mahoney (2011) for a small (*n* = 4) mixed archaeological sample of dm_1_.

The range of prenatal enamel formation times that we observed in our present‐day and archaeological samples was much greater than that reported previously for deciduous molars. When our data are recalculated as gestational weeks, the earliest initiation of first molars occurred during the 17th gestational week. Sunderland et al. (1987) reported the earliest onset of mineralization in the 16th gestational week for dm1 dentin. However, the range of dm1 initiation times in our study is much wider than that reported by Sunderland et al. (1987). They reported initiation between gestational Weeks 16 and 19 for dm1, and Weeks 20 to 22 for dm2 (Sunderland et al. 1987). In our study, initiation occurred between gestational weeks 17 and 29 for dm1, and 20 to 35 weeks for dm2. The wide range of initiation times we observed in first molars (87 to 167 days before birth) is similar to and overlaps with the prenatal range of 88 to 201 days before birth reported by Magri et al. (2024) for *n* = 30 Italian Medieval deciduous first molars. Similarly, a wide range of prenatal initiation times has been reported for present‐day deciduous canines (Dean et al. 2020).

Our 95% confidence interval (CI) that initiation occurred between 135 and 124 days before birth for first molars from the present‐day sample, is close to the estimated CI of 146 to 135 days calculated by Birch and Dean (2014) from regression equations applied to *n* = 10 thin sections of present‐day dm1. Our 95% CI of 95 to 87 days for present‐day dm2 samples is less than the 95% CI estimate of 122 to 113 days by Birch and Dean (2014) for this tooth type, though our range of dm2 initiation times encompasses their estimate.

#### 4.1.2 Between Present‐Day and Archaeological Samples

While there was substantial overlap in the total Cu‐Ft of all populations, between a third of the second molars and a quarter of first molars from the combined archaeological periods extended below the lower‐most total Cu‐Ft of the present‐day samples (Figure 4). On average, molar enamel cusps from the archaeological periods were complete from 3.1 to 2.5 months ahead of present‐day samples (Figure 6). These differences between present‐day and archaeological populations were a product of mainly the postnatal period, not the prenatal period of enamel growth (Figure 7C).

Differences between samples could be less marked when individual populations were considered. Upper second molars and lower first molars of Pacific Island peoples in particular formed over a period that was similar to the Cu‐Ft of the archaeological periods. This was typical of the variation between present‐day and archaeological populations in that when it was absent, or present and significant, it was not specific to one tooth type. For example, Iron Age upper dm2 differed significantly compared to all present‐day samples but the lower dm1 did not. Conversely, Roman upper dm1 differed to all present‐day samples, but the lower dm2 only differed to the Canadian sample.

### 4.2 Sources of Variation in Enamel Formation Time

#### 4.2.1 Enamel Growth Rates

Temporal variation in DSRs has been reported whereby rates in certain regions of deciduous incisors from Roman Italy, and permanent M1 from Anglo‐Saxon and Roman United Kingdom, were faster compared to present‐day samples from the United Kingdom (Nava, Coppa, et al. 2017; Aris, Mahoney, O'Hara, and Deter 2020). Our findings for deciduous molars are consistent with those studies. Our mean DSRs lay between 4.40 and 5.30 μm for mid and outer enamel regions in the archaeological samples of deciduous molars. These mean DSRs are greater than the mean DSR of 3.80 μm reported previously by McFarlane et al. (2021) for present‐day samples. However, when just the archaeological samples are compared, there was no consistent temporal variation in secretion rates from the more ancient Iron Age to the more recent Medieval period.

**FIGURE 11:**
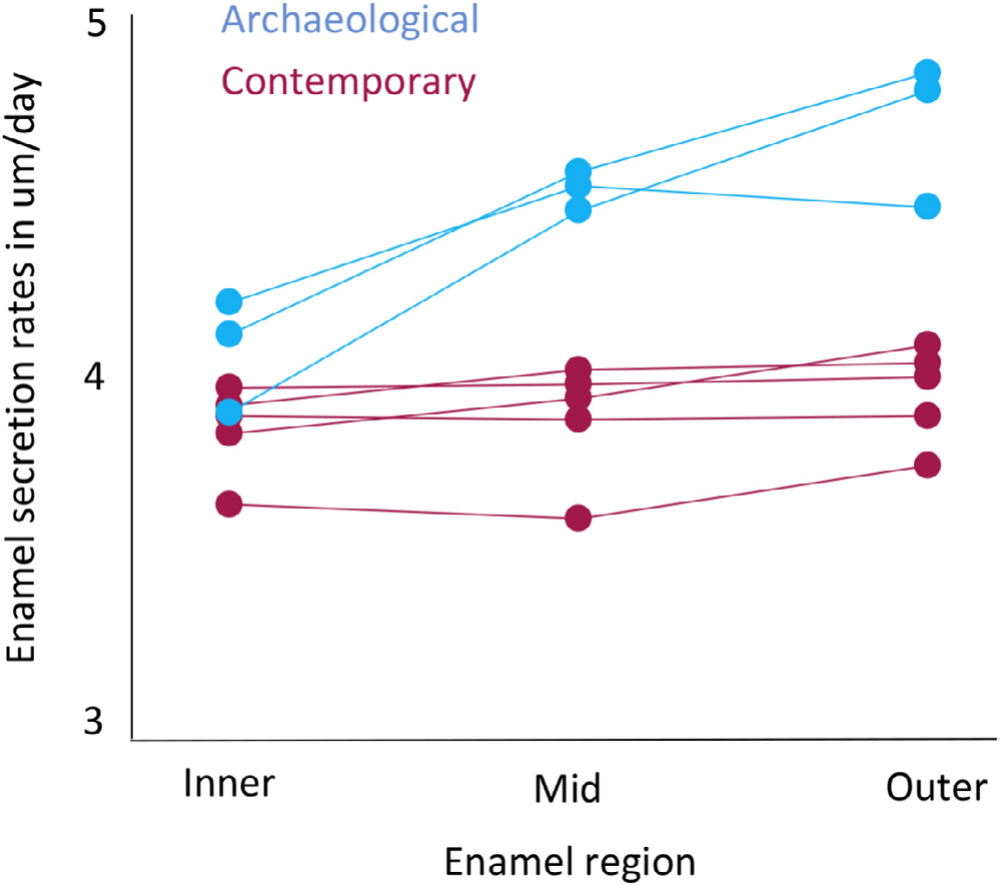
Comparing deciduous molar enamel daily secretion rates. Mean rates for archaeological samples of upper and lower molars combined and taken from Table 5. Secretion rates for present‐day NZ‐Europeans, British, Canadian, French and Swedish deciduous molars taken from McFarlane et al. (2021).

Variation in mean cuspal DSRs between inner and outer enamel regions in our archaeological samples produces a growth trajectory that differs from that observed for present‐day deciduous molars (Figure 11). In present‐day molars, mean DSRs change only slightly between these regions (McFarlane et al. 2021). Our archaeological samples of deciduous molars grow differently, as ameloblasts secrete new matrix slowly nearer the EDJ but then these cells start to accelerate producing rates that lie above the mean rates reported for present‐day samples. These slow‐to‐fast rates produce a steeper gradient in the trajectory of enamel secretion, which together with higher initial extension rates, likely contributes to shorter periods of enamel formation in the archaeological samples, compared to present‐day deciduous molars. For example, when using a mean value from the mid‐enamel region from our Table 5, a 200 μm prism length would form in about 44 days in archaeological samples. Using previously published data for the same region in present‐day deciduous molars (McFarlane et al. 2021) would produce a 200 μm prism length over 54 days. The difference is not great, but the effect would likely be cumulative throughout the formation period, and this would be augmented by the higher extension rates we observed in the archaeological samples (Table 6).

#### 4.2.2 Enamel Thickness

Average enamel thickness varied only slightly and inconsistently when compared between all samples. Thus, the average straight‐line thickness between the EDJ and the outer enamel surface was similar in the archaeological and present‐day deciduous molars, but these same populations differed in that the enamel formed over a shorter post‐natal period with an accelerated rate of growth in the archaeological samples.

Prior research has reported that there was no consistent temporal trend in the relative and lateral enamel thickness of permanent first molars from archaeological periods in the United Kingdom (Aris, Mahoney, O'Hara, and Deter 2020). Our findings for deciduous enamel thickness are broadly similar to those reported for permanent first molars, and differ only slightly in that our predicted differences in enamel thickness between Roman and present‐day samples, based upon permanent first molars, were not present in deciduous molars. This may simply reflect our methodology in that we examined AET and did not consider variation in lateral enamel thickness, or the regional distribution of enamel over the crown surface (e.g., Le Luyer and Bayle 2017; Aris, Mahoney, O'Hara, and Deter 2020). Alternatively, the temporary nature of deciduous molars might be a contributing factor here. Deciduous molars are typically shed between the ages of eight and 13 years. Pampush et al. (2013) have shown a relationship between lifetime dietary wear and enamel thickness amongst primate species. If these findings are extrapolated here, then perhaps variation in deciduous molar mean AET is less obvious because this tooth type, unlike permanent teeth, does not have to function over a lifetime.

#### 4.2.3 The Pace of Deciduous Dental Development

The Pacific Island children were more similar to our archaeological samples than the other modern groups in that their deciduous molars formed over a shorter period with a faster growth rate. Relatively accelerated deciduous molar development amongst Pacific Island children is consistent with their advanced permanent molar mineralization and early schedule of tooth emergence (Te Moananui et al. 2008). This schedule makes sense when considered alongside their generally faster pace of somatic growth that includes advanced skeletal (vertebrae) growth, greater height for certain ages, as well as a relatively early age at pubertal onset (Petelo 2011; Rush et al. 2016; Baylis et al. 2024; Kubo et al. 2024).

The similar deciduous molar development of the Pacific Island peoples and our archaeological samples could reflect a similar pace to their somatic growth. Teeth develop as part of a growing organism, which is seen across modern primate species generally where dental development is linked to the duration of somatic growth and the pace of life history including body size and age at maturity (Smith 1989, 2024; Smith et al. 1994). Life history theory explains how organisms allocate limited energy between growth, reproduction, and survival, with ecological pressures forcing trade‐offs between these functions (Hill and Kaplan 1999; Stearns 2000). Benefits of early maturation include shorter exposure to juvenile mortality risks before first reproduction (Stearns 2000). The more developmentally advanced an infant is at birth, the more likely they are to survive early infections (Longo et al. 2014; Simchen et al. 2000). Faster growth can be a response to high infant and child mortality rates associated with infectious and parasitic diseases (Thomas et al. 2004; Walker et al. 2006).

High mortality rates may partly explain why the archaeological samples of deciduous molars formed over a shorter period. High extrinsic mortality during infancy and childhood exerts strong selective pressure for accelerated development (Kuzawa and Bragg 2012), as fitness is highly selective to shifts in early survival rates (Jones 2009; Volk and Atkinson 2008). Historically, infant and child mortality rates could be high with approximately 30% of infants dying in their first year, and nearly half dying before 15 years of age (Volk and Atkinson 2013). In 16th and 17th century AD England, it is estimated that 27% to 37% of infants died (Matossian 1985; Orme 1995). Over the last 150 years however, most industrialized countries have seen dramatic declines, with modern European infant mortality now usually below 4 per 1000 births (Volk and Atkinson 2008, 2013; Woods 1993; World Bank Open Data 2023). Under this scenario, the accelerated deciduous molar development of our archaeological samples may have been an adaptive response to elevated mortality rates, compared with modern declines in mortality that released selective pressures for rapid growth (Kuzawa and Bragg 2012; Volk and Atkinson 2008). Thus, deciduous molar development amongst the Medieval, Roman and Iron Age children may reflect a trade‐off, whereby high infant mortality favored faster growth to increase the chances of early survival. This assumes of course that the relatively early postnatal formation of their molar crowns facilitated an earlier schedule of tooth emergence, similar to that which has been observed for the deciduous canines of the Baka pygmies (Tiwa et al. 2024). One does not however have to follow the other, if there was for example a shift in the root development of our archaeological samples. Early crown formation might combine with an extended period of root formation that could ultimately delay tooth emergence times. The proportion of root that is formed can vary greatly as deciduous crowns start to emerge (Liversidge and Molleson 2017). Future studies might test these different scenarios.

The benefits of early maturation related to reduced exposure to juvenile mortality might have been one contributing selective pressure to the variation we observed amongst the present‐day samples. Pacific Island nations have higher infant mortality rates, 22 per 1000 births (World Bank Open Data 2023), which may mean that selection pressures favoring accelerated early growth may not have diminished to the same extent as in other modern populations with lower infant and child mortality rates.

### 4.3 Applications of Deciduous Molar Formation Time

#### 4.3.1 Tooth Type

Differences in Cu‐Ft between upper and lower second deciduous molars indicate that these tooth types should be treated separately in analyses of formation time and are not likely to be an accurate substitute for each other. Upper and lower first molars are closer in mean formation times, even though the upper molar is slightly larger, and can probably be substituted for each other. The additional time required to form enamel covering the tubercle of Zuckerkandl in lower dm_1_ is likely responsible for the similarity in formation time between this molar and its isomere (Mahoney 2011).

#### 4.3.2 Present‐Day Analogues and Estimating Age‐at‐Death

Deciduous crown formation times that have been related to chronological age are frequently drawn upon to estimate age‐at‐death for human infant and juvenile skeletons recovered from archaeological contexts (e.g., Moorrees et al. 1963). In our study, between 34% (dm2) and 25% (dm1) of the total Cu‐Ft from the archaeological sample extended below those from the present day. For upper dm1, the difference increased greatly, as 60% of the post‐natal Cu‐Fts from the archaeological samples extended below the lowermost range from the present‐day samples. This lowered range and the lowered mean values imply that tooth formation times from some of our present‐day populations would not have been an accurate proxy from which to estimate age‐at‐death for the archaeological samples in our study. This differs from our finding for Pacific Island peoples, and the upper dm2 of this population in particular, which was much closer to the formation time of the archaeological samples. It seems feasible therefore that studies could start to distinguish between present‐day samples to identify which is the most appropriate analogue for estimating age‐at‐death when working with archaeological materials. Histology could make a useful contribution here by estimating biological age. Biological age does not equal chronological age but there is a very strong correlation between the two when using histology (Smith et al. 2006; Antoine et al. 2009). Histology could be applied to a small sample of archaeological skeletons where there is no record of chronological age (Boyde 1963), to gain insight into the schedule of dental development, which could then inform the application of the most appropriate modern‐day dental growth standard.

#### 4.3.3 Postnatal Formation Time

Differences in formation times between the populations examined here relate mainly to the duration of the postnatal period. This period of tooth formation after birth may prove to be of particular interest in studies of ontogeny inferred from dental histology, not least because it can be accessed histologically in teeth that are heavily worn. The neonatal line is typically preserved in the lateral or cervical enamel of deciduous incisors, canines, and first molars (Mahoney 2015). Thus, postnatal Cu‐Ft can often be calculated in these deciduous teeth when the cuspal/occlusal enamel is worn or has been completely removed.

### 4.4 Lateral Enamel Scaling Proportions

Formation times calculated from our extension rates methodology for segments two, three and four of the EDJ, were similar when compared to those calculated by others that have used Retzius lines or perikymata (Appendix 8). The formation time for each of these segments, recalculated as a percentage of the total time taken to form the height of the EDJ in Table 8 can be utilized to scale the lateral enamel formation time of deciduous molars.

It took a proportionally greater time to form the first segment of enamel when using Retzius lines or perikymata, compared to our extension rate methodology (Appendix 8). The differences in the time taken to form segment one when compared between the methodologies are probably due to the thick M1 cuspal enamel relative to the cervical half of the crown. In the cuspal enamel, ameloblasts take longer to reach, and form, the outer enamel surface. This likely creates a time lag between the two processes that is accentuated by the rapid initial EDJ extension, resulting in outer enamel taking proportionally longer to form. Further towards the cervix, extension rates slow and M1 enamel becomes thinner so ameloblasts reach and form the surface more quickly and differences between the EDJ and outer lateral surface are reduced.

## 5 Conclusions

This study examined histologically derived modern human enamel cusp formation times for deciduous first and second molars from present day and archaeological populations. On average, the duration of prenatal enamel formation was generally similar across all samples for each tooth type. The archaeological populations had a shorter postnatal period of enamel formation that was facilitated by a faster underlying rate of enamel growth, when compared to the present‐day samples.

## Author Contributions

**Patrick Mahoney:** conceptualization, methodology, data curation, investigation, formal analysis, funding acquisition, writing – review and editing, writing – original draft. **Gina McFarlane:** methodology, investigation, writing – original draft, writing – review and editing, formal analysis. **Petrina Barnard:** methodology, writing – review and editing. **Rosie Pitfield:** methodology, writing – review and editing. **Mackie C. O'Hara:** writing – review and editing, investigation, formal analysis. **Alfredo Coppa:** investigation, writing – review and editing. **Carmen Esposito:** investigation, writing – review and editing. **Alessandra Sperduti:** investigation, writing – review and editing. **Chris Deter:** methodology, investigation, writing – review and editing. **Alessia Nava:** methodology, investigation, writing – original draft, writing – review and editing. **Carolina Loch:** conceptualization, investigation, funding acquisition, writing – original draft, writing – review and editing.

## Conflicts of Interest

The authors declare no conflicts of interest.

## Supporting information


**Appendix 1** Calculation for scaling lateral enamel formation time.

**Appendix 2** TOTAL CU‐FT. Pairwise comparisons.

**Appendix 3** Postnatal formation times. Pairwise comparisons.

**Appendix 4** Prenatal formation times. Pairwise comparisons.

**Appendix 5** Extension rates for DM1.

**Appendix 6** Extension rates for DM2.

**Appendix 7** Average enamel thickness. Pairwise comparisons.

**Appendix 8** Test of lateral enamel scaling proportions.

## Data Availability

The data that support the findings of this study are available from the corresponding author upon reasonable request.
