# Supplementary material for: Variation in Modern Human Deciduous Molar Enamel Formation Time
Source: Am J Biol Anthropol. 2025 Nov 14;188(3):e70156. doi: 10.1002/ajpa.70156 (PMC12616781; doi:10.1002/ajpa.70156)
Supplement: Supplementary file 1 — Appendix 1 Calculation for scaling lateral enamel formation time. [file AJPA-188-e70156-s003.pdf]

APPENDIX 1.

Calculation for scaling lateral enamel formation time

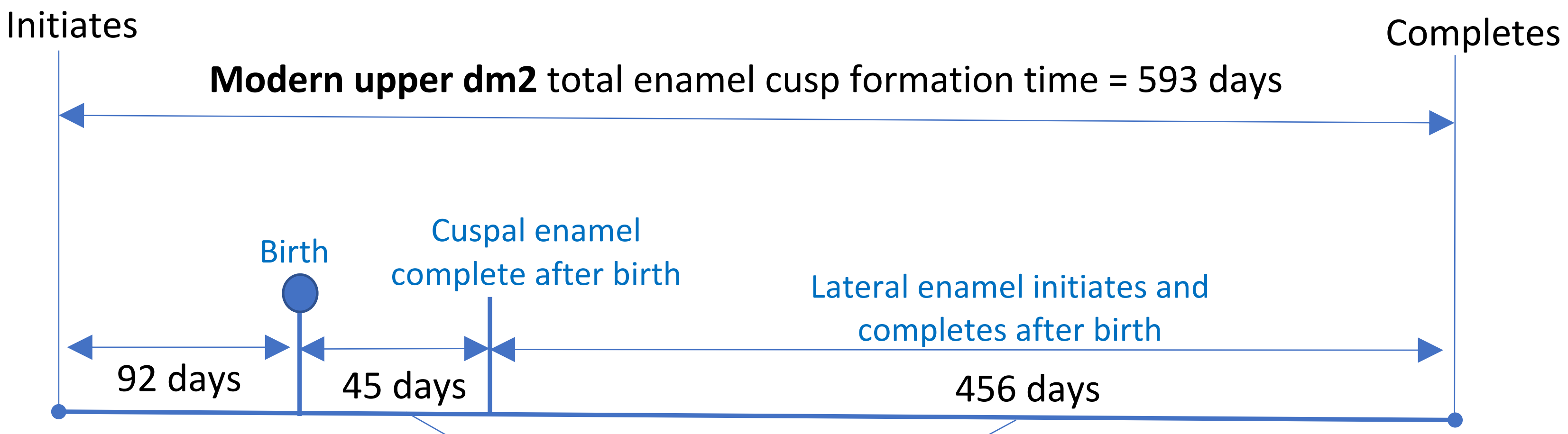

**Segment**	Table 7 percentages	Segment formation time in days	Cumulative time in days	Postnatal cuspal enamel formation time in days	Postnatal lateral enamel formation time in days
**1**	10.87% of 456 days =	50			
**2**	20.42% of 456 days =	93	143	+ 45 =	188
**3**	29.59% of 456 days =	135	278	+ 45 =	323
**4**	39.12% of 456 days =	178	456	+ 45 =	501

These numbers are used in:

- Table 3 upper dm2 (all modern combined)
- Figure 6

APPENDIX 1.

Calculation for scaling lateral enamel formation time

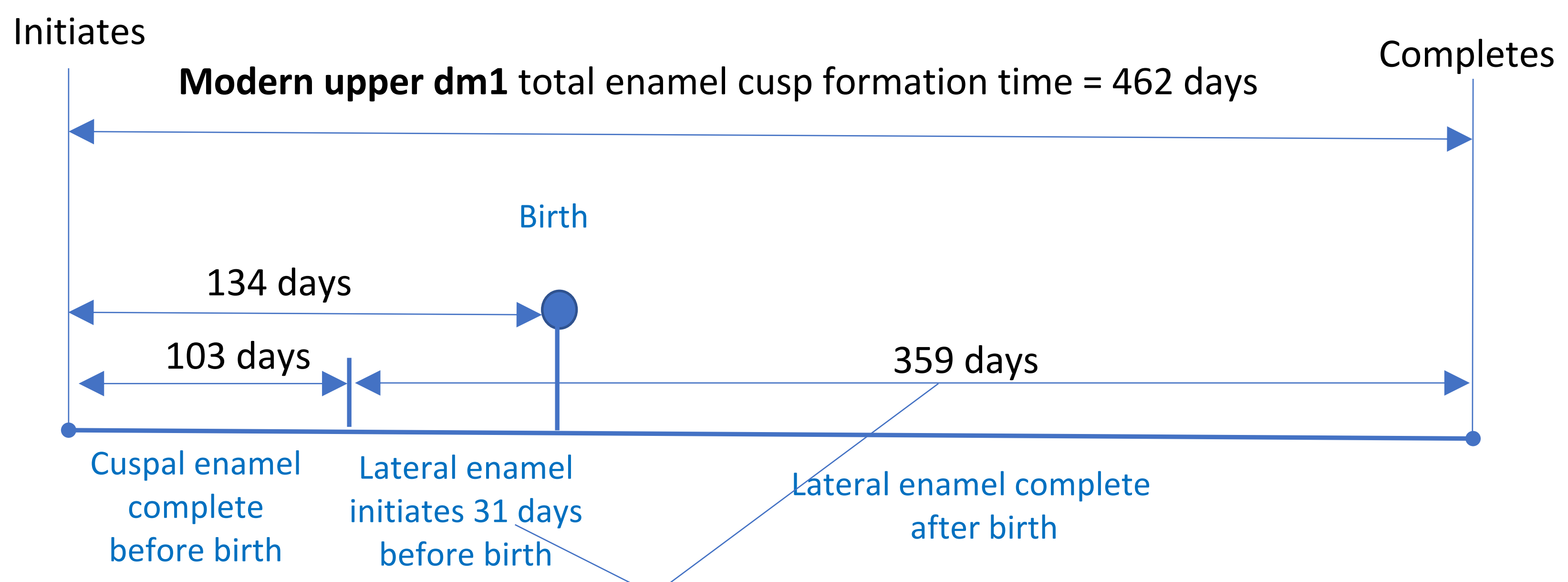

**Segment**	Table 7 percentages	Segment formation time in days	Cumulative time in days	Prenatal lateral enamel formation time in days	Postnatal lateral enamel formation time in days
**1**	12.62% of 359 days =	45			
**2**	20.48% of 359 days =	73	118	- 31 =	87
**3**	29.11% of 359 days =	105	223	- 31 =	192
**4**	37.79% of 359 days =	136	359	- 31 =	328

These numbers are used in:

- Table 3 upper dm1 (all modern combined)
- Figure 6
