# Supplementary material for: Variation in Modern Human Deciduous Molar Enamel Formation Time
Source: Am J Biol Anthropol. 2025 Nov 14;188(3):e70156. doi: 10.1002/ajpa.70156 (PMC12616781; doi:10.1002/ajpa.70156)
Supplement: Supplementary file 2 — Appendix 2 TOTAL CU‐FT. Pairwise comparisons. [file AJPA-188-e70156-s007.pdf]

APPENDIX 2.

TOTAL CU-FT. Pairwise comparisons

Present-day. **UPPER DM2 Within-samples**

	Test Statistic	Std. Error	Std. Test Statistic	Sig.	Adj. Sig.[a]
Pacific-Maori	12.359	9.269	1.333	.182	1.000
Pacific-NZ	20.084	7.963	2.522	.012	.117
**Pacific-British**	**22.045**	**7.834**	**2.814**	**.005**	**.049**
**Pacific-Canadian**	**-26.059**	**9.269**	**-2.811**	**.005**	**.049**
Maori-NZ	7.725	8.216	.940	.347	1.000
Maori-British	9.686	8.091	1.197	.231	1.000
Maori-Canadian	-13.700	9.487	-1.444	.149	1.000
NZ-British	1.961	6.554	.299	.765	1.000
NZ-Canadian	-5.975	8.216	-.727	.467	1.000
British-Canadian	-4.014	8.091	-.496	.620	1.000

Each row tests the null hypothesis that the distributions of the two populations are the same. Asymptotic significances (2-sided tests) are displayed. The significance level is p=0.050. [a]Significance values have been adjusted by the Bonferroni correction for multiple tests. Significant results are in bold.

Present-day. **LOWER DM2 Within-samples**

Sample 1-Sample 2	Test Statistic	Std. Error	Std. Test Statistic	Sig.	Adj. Sig.[a]
Pacific-British	4.614	6.907	.668	.504	1.000
Pacific-NZ	9.313	6.436	1.447	.148	1.000
Pacific-Canadian	-10.750	8.028	-1.339	.181	1.000
Pacific-Maori	13.100	7.051	1.858	.063	.632
British-NZ	-4.699	5.822	-.807	.420	1.000
British-Canadian	-6.136	7.544	-.813	.416	1.000
British-Maori	-8.486	6.495	-1.307	.191	1.000
NZ-Canadian	-1.438	7.116	-.202	.840	1.000
NZ-Maori	-3.788	5.992	-.632	.527	1.000
Canadian-Maori	2.350	7.676	.306	.759	1.000

APPENDIX 2.
TOTAL CU-FT. Pairwise comparisons

Archaeological. **UPPER DM2 Within-samples**

	Test Statistic	Std. Error	Std. Test Statistic	Sig.	Adj. Sig.[a]
Iron Age-Rome	1.419	5.102	.278	.781	1.000
Iron Age-Medieval	7.873	3.718	2.117	.034	.103
Rome-Medieval	6.454	5.240	1.232	.218	.654

Archaeological. **LOWER DM2 Within-samples**

	Test Statistic	Std. Error	Std. Test Statistic	Sig.	Adj. Sig.[a]
Medieval-Iron Age	-3.472	4.149	-.837	.403	1.000
Medieval-Rome	-5.972	4.149	-1.440	.150	.450
Iron Age-Rome	2.500	5.081	.492	.623	1.000

APPENDIX 2.
TOTAL CU-FT. Pairwise comparisons

Present-day vs archaeological. **UPPER DM2**

Sample 1-Sample 2	Test Statistic	Std. Error	Std. Test Statistic	Sig.	Adj. Sig.[a]
Rome-Pacific	31.109	16.737	1.859	.063	1.000
Rome-Maori	45.550	16.996	2.680	.007	.206
**Rome-NZ**	**53.725**	**15.515**	**3.463**	**<.001**	**.015**
**Rome-British**	**56.905**	**15.374**	**3.701**	**<.001**	**.006**
**Rome-Canadian**	**61.050**	**16.996**	**3.592**	**<.001**	**.009**
Iron Age-Pacific	29.003	12.154	2.386	.017	.477
**Iron Age-Maori**	**43.444**	**12.509**	**3.473**	**<.001**	**.014**
**Iron Age-NZ**	**51.619**	**10.408**	**4.960**	**<.001**	**.000**
**Iron Age-British**	**54.798**	**10.196**	**5.375**	**<.001**	**.000**
**Iron Age-Canadian**	**58.944**	**12.509**	**4.712**	**<.001**	**.000**
Medieval-Pacific	10.678	12.712	.840	.401	1.000
Medieval-Maori	25.119	13.052	1.925	.054	1.000
Medieval-NZ	33.294	11.055	3.012	.003	.073
**Medieval-British**	**36.474**	**10.855**	**3.360**	**<.001**	**.022**
**Medieval-Canadian**	**40.619**	**13.052**	**3.112**	**.002**	**.050**

Each row tests the null hypothesis that the distributions of the two populations are the same. Asymptotic significances (2-sided tests) are displayed. The significance level is p=0.050. [a]Significance values have been adjusted by the Bonferroni correction for multiple tests. Significant results are in bold.

APPENDIX 2.
TOTAL CU-FT. Pairwise comparisons

Present-day vs archaeological. **LOWER DM2**

Sample 1-Sample 2	Test Statistic	Std. Error	Std. Test Statistic	Sig.	Adj. Sig.
Medieval-Pacific	26.299	9.996	2.631	.009	.238
**Medieval-British**	**32.543**	**9.003**	**3.615**	**<.001**	**.008**
**Medieval-NZ**	**39.174**	**8.083**	**4.846**	**<.001**	**.000**
**Medieval-Canadian**	**41.694**	**11.090**	**3.760**	**<.001**	**.005**
**Medieval-Maori**	**42.711**	**9.278**	**4.603**	**<.001**	**.000**
Iron Age-Pacific	22.354	12.705	1.759	.078	1.000
Iron Age-British	28.598	11.939	2.395	.017	.465
**Iron Age-NZ**	**35.229**	**11.262**	**3.128**	**.002**	**.049**
Iron Age-Canadian	37.750	13.582	2.779	.005	.152
**Iron Age-Maori**	**38.767**	**12.148**	**3.191**	**.001**	**.040**
Rome-Pacific	20.188	12.705	1.589	.112	1.000
Rome-British	26.432	11.939	2.214	.027	.752
Rome-NZ	33.063	11.262	2.936	.003	.093
**Rome-Canadian**	**35.583**	**13.582**	**2.620**	**.009**	**.046**
Rome-Maori	34.600	12.148	3.013	.003	.072

APPENDIX 2.
TOTAL CU-FT. Pairwise comparisons

Present-day. **UPPER DM1 Within-samples**

Sample 1-Sample 2	Test Statistic	Std. Error	Std. Test Statistic	Sig.	Adj. Sig.[a]
Maori-NZ	.464	5.674	.082	.935	1.000
Maori-British	7.506	5.391	1.392	.164	.983
Maori-Canadian	-9.396	5.522	-1.702	.089	.533
NZ-British	7.042	5.868	1.200	.230	1.000
NZ-Canadian	-8.932	5.988	-1.492	.136	.815
British-Canadian	-1.890	5.720	-.330	.741	1.000

Present-day. **LOWER DM1 Within-samples**

	Test Statistic	Std. Error	Std. Test Statistic	Sig.	Adj. Sig.[a]
Pacific-British	10.170	5.434	1.871	.061	.368
Pacific-Maori	10.451	5.958	1.754	.079	.476
Pacific-NZ	13.108	5.698	2.301	.021	.128
British-Maori	-.282	5.239	-.054	.957	1.000
British-NZ	-2.938	4.940	-.595	.552	1.000
Maori-NZ	2.657	5.511	.482	.630	1.000

Archaeological **LOWER DM1 Within-samples**[1]

	Test Statistic	Std. Error	Std. Test Statistic	Sig.	Adj. Sig.[a]
**Rome-Medieval**	**14.248**	**4.848**	**2.939**	**.003**	**.010**
**Rome-Iron Age**	**-15.048**	**5.806**	**-2.592**	**.010**	**.029**
Medieval-Iron Age	-.800	6.389	-.125	.900	1.000

1=Upper dm1 Medieval compared to Roman samples (p=0.088)

APPENDIX 2.

TOTAL CU-FT. Pairwise comparisons

Present-day vs archaeological **UPPER dm1.**

	Test Statistic	Std. Error	Std. Test Statistic	Sig.	Adj. Sig.[a]
**Rome-Maori**	**31.214**	**10.450**	**2.987**	**.003**	**.042**
**Rome-NZ**	**31.650**	**10.987**	**2.881**	**.004**	**.050**
**Rome-British**	**39.625**	**10.677**	**3.711**	**<.001**	**.003**
**Rome-Canadian**	**41.636**	**10.819**	**3.848**	**<.001**	**.002**
Medieval-Maori	16.803	7.239	2.321	.020	.304
Medieval-NZ	17.238	7.994	2.156	.031	.466
**Medieval-British**	**25.213**	**7.563**	**3.334**	**<.001**	**.013**
**Medieval-Canadian**	**27.225**	**7.762**	**3.508**	**<.001**	**.007**

Present-day vs archaeological **LOWER dm1.**

Sample 1-Sample 2	Test Statistic	Std. Error	Std. Test Statistic	Sig.	Adj. Sig.[a]
Rome-Pacific	31.375	10.935	2.869	.004	.086
**Rome-British**	**47.036**	**8.929**	**5.268**	**<.001**	**.000**
**Rome-Maori**	**50.667**	**10.452**	**4.848**	**<.001**	**.000**
**Rome-NZ**	**54.091**	**9.706**	**5.573**	**<.001**	**.000**
Medieval-Pacific	14.042	11.942	1.176	.240	1.000
Medieval-British	29.702	10.136	2.930	.003	.071
Medieval-Maori	33.333	11.501	2.898	.004	.079
**Medieval-NZ**	**36.758**	**10.828**	**3.395**	**<.001**	**.014**
Iron Age-Pacific	11.764	13.254	.888	.375	1.000
Iron Age-British	27.425	11.654	2.353	.019	.391
Iron Age-Maori	31.056	12.859	2.415	.016	.330
Iron Age-NZ	34.480	12.260	2.812	.005	.103
