# Supplementary material for: Variation in Modern Human Deciduous Molar Enamel Formation Time
Source: Am J Biol Anthropol. 2025 Nov 14;188(3):e70156. doi: 10.1002/ajpa.70156 (PMC12616781; doi:10.1002/ajpa.70156)
Supplement: Supplementary file 3 — Appendix 3 Postnatal formation times. Pairwise comparisons. [file AJPA-188-e70156-s008.pdf]

APPENDIX 3

POSTNATAL FORMATION TIMES. Pairwise comparisons

UPPER DM1 Within-samples

Sample 1-Sample 2	Test Statistic	Std. Error	Std. Test Statistic	Sig.	Adj. Sig.[a]
NZ-Maori	-2.095	4.414	-.475	.635	1.000
NZ-Canadian	-4.595	4.414	-1.041	.298	1.000
NZ-British	7.452	4.414	1.688	.091	.548
Maori-Canadian	-2.500	4.241	-.590	.556	1.000
Maori-British	5.357	4.241	1.263	.206	1.000
Canadian-British	2.857	4.241	.674	.500	1.000

Each row tests the null hypothesis that the Sample 1 and Sample 2 distributions are the same. Asymptotic significances (2-sided tests) are displayed. The significance level is .050. a. Significance values have been adjusted by the Bonferroni correction for multiple tests.

LOWER DM1 Within-samples

Sample 1-Sample 2	Test Statistic	Std. Error	Std. Test Statistic	Sig.	Adj. Sig.[a]
Pacific-NZ	8.634	4.704	1.836	.066	.399
Pacific-Maori	10.214	4.858	2.103	.036	.213
Pacific-British	10.460	4.580	2.284	.022	.134
NZ-Maori	-1.580	4.704	-.336	.737	1.000
NZ-British	1.826	4.416	.414	.679	1.000
Maori-British	.246	4.580	.054	.957	1.000

LOWER DM1 Within-samples

Sample 1-Sample 2	Test Statistic	Std. Error	Std. Test Statistic	Sig.	Adj. Sig.[a]
Rome-Medieval	8.167	4.368	1.870	.062	.185
Rome-Iron Age	-13.000	5.295	-2.455	.014	.062
Medieval-Iron Age	-4.833	5.833	-.829	.407	1.000

UPPER DM1 ARCHAEO MEDIEVAL VS ROME (U=2.420; p=0.120)

APPENDIX 3

POSTNATAL FORMATION TIMES. Pairwise comparisons

UPPER DM1 Between-samples

	Test Statistic	Std. Error	Std. Test Statistic	Sig.	Adj. Sig.[a]
Rome-Pacific	23.634	9.158	2.581	.010	.207
**Rome-NZ**	**38.785**	**8.708**	**4.454**	**<.001**	**.000**
**Rome-British**	**40.292**	**8.341**	**4.831**	**<.001**	**.000**
**Rome-Maori**	**40.419**	**9.158**	**4.414**	**<.001**	**.000**
Medieval-Iron Age	-6.577	10.090	-.652	.514	1.000
Medieval-Pacific	14.577	10.090	1.445	.149	1.000
**Medieval-NZ**	**29.729**	**9.683**	**3.070**	**.002**	**.045**
**Medieval-British**	**31.236**	**9.355**	**3.339**	**<.001**	**.018**
**Medieval-Maori**	**31.363**	**10.090**	**3.108**	**.002**	**.039**
Iron Age-Pacific	8.000	11.340	.705	.481	1.000
Iron Age-NZ	23.152	10.980	2.109	.035	.735
Iron Age-British	24.659	10.691	2.306	.021	.443
Iron Age-Maori	24.786	11.340	2.186	.029	.606
NZ-British	1.507	10.308	.146	.884	1.000
NZ-Maori	-1.634	10.980	-.149	.882	1.000
British-Maori	-.127	10.691	-.012	.991	1.000

LOWER DM1 Between-samples

	Test Statistic	Std. Error	Std. Test Statistic	Sig.	Adj. Sig.[a]
Rome-Medieval	11.650	7.086	1.644	.100	1.000
Rome-NZ	15.583	7.731	2.016	.044	.658
Rome-Maori	18.679	7.507	2.488	.013	.193
**Rome-Canadian**	**21.607**	**7.507**	**2.878**	**.004**	**.050**
**Rome-British**	**24.893**	**7.507**	**3.316**	**<.001**	**.014**
Medieval-NZ	3.933	6.185	.636	.525	1.000
Medieval-Maori	7.029	5.903	1.191	.234	1.000
Medieval-Canadian	9.957	5.903	1.687	.092	1.000
Medieval-British	13.243	5.903	2.244	.025	.373
NZ-Maori	-3.095	6.664	-.464	.642	1.000
Canadian-British	3.286	6.402	.513	.608	1.000

APPENDIX 3

POSTNATAL FORMATION TIMES. Pairwise comparisons

UPPER DM2 Between-samples

	Test Statistic	Std. Error	Std. Test Statistic	Sig.	Adj. Sig.[a]
Rome-Iron Age	-.573	10.352	-.055	.956	1.000
Rome-Medieval	10.454	10.100	1.035	.301	1.000
Rome-Pacific	21.729	11.239	1.933	.053	1.000
Rome-British	26.400	12.139	2.175	.030	.830
Rome-Maori	26.633	10.706	2.488	.013	.360
**Rome-NZ**	**34.856**	**10.706**	**3.256**	**.001**	**.032**
**Rome-Canadian**	**37.300**	**11.239**	**3.319**	**<.001**	**.025**
Iron Age-Medieval	9.881	7.863	1.257	.209	1.000
Iron Age-Pacific	21.156	9.280	2.280	.023	.633
Iron Age-British	25.827	10.352	2.495	.013	.353
**Iron Age-Maori**	**26.061**	**8.627**	**3.021**	**.003**	**.049**
**Iron Age-NZ**	**34.283**	**8.627**	**3.974**	**<.001**	**.002**
**Iron Age-Canadian**	**36.727**	**9.280**	**3.958**	**<.001**	**.002**
Medieval-Pacific	11.275	8.998	1.253	.210	1.000
Medieval-British	15.946	10.100	1.579	.114	1.000
Medieval-Maori	16.179	8.323	1.944	.052	1.000
Medieval-NZ	24.402	8.323	2.932	.003	.064
**Medieval-Canadian**	**26.846**	**8.998**	**2.984**	**.003**	**.050**
Pacific-British	4.671	11.239	.416	.678	1.000
Pacific-Maori	4.905	9.673	.507	.612	1.000
Pacific-NZ	13.127	9.673	1.357	.175	1.000
Pacific-Canadian	-15.571	10.259	-1.518	.129	1.000
British-Maori	-.233	10.706	-.022	.983	1.000
British-NZ	-8.456	10.706	-.790	.430	1.000
British-Canadian	-10.900	11.239	-.970	.332	1.000
Maori-NZ	8.222	9.048	.909	.363	1.000
Maori-Canadian	-10.667	9.673	-1.103	.270	1.000
NZ-Canadian	-2.444	9.673	-.253	.800	1.000

APPENDIX 3

POSTNATAL FORMATION TIMES. Pairwise comparisons

LOWER DM2 Between-samples

	Test Statistic	Std. Error	Std. Test Statistic	Sig.	Adj. Sig.[a]
Medieval-Iron Age	-2.050	9.091	-.226	.822	1.000
Medieval-Rome	-4.750	9.091	-.523	.601	1.000
Medieval-British	17.750	7.873	2.255	.024	.676
Medieval-Pacific	17.964	8.179	2.196	.028	.786
**Medieval-NZ**	**27.250**	**7.107**	**3.834**	**<.001**	**.004**
Medieval-Canadian	28.875	9.819	2.941	.003	.092
**Medieval-Maori**	**35.083**	**8.571**	**4.093**	**<.001**	**.001**
Iron Age-Rome	2.700	10.497	.257	.797	1.000
Iron Age-British	15.700	9.462	1.659	.097	1.000
Iron Age-Pacific	15.914	9.718	1.638	.102	1.000
Iron Age-NZ	25.200	8.835	2.852	.004	.121
Iron Age-Canadian	26.825	11.134	2.409	.016	.448
**Iron Age-Maori**	**33.033**	**10.050**	**3.287**	**.001**	**.028**
Rome-British	13.000	9.462	1.374	.169	1.000
Rome-Pacific	13.214	9.718	1.360	.174	1.000
Rome-NZ	22.500	8.835	2.547	.011	.304
Rome-Canadian	24.125	11.134	2.167	.030	.847
**Rome-Maori**	**30.333**	**10.050**	**3.018**	**.003**	**.049**
British-Pacific	-.214	8.590	-.025	.980	1.000
British-NZ	-9.500	7.576	-1.254	.210	1.000
British-Canadian	-11.125	10.164	-1.095	.274	1.000
British-Maori	-17.333	8.964	-1.934	.053	1.000
Pacific-NZ	9.286	7.894	1.176	.239	1.000
Pacific-Canadian	-10.911	10.403	-1.049	.294	1.000
Pacific-Maori	17.119	9.234	1.854	.064	1.000
NZ-Canadian	-1.625	9.583	-.170	.865	1.000
NZ-Maori	-7.833	8.299	-.944	.345	1.000
Canadian-Maori	6.208	10.714	.579	.562	1.000
