# Supplementary material for: Variation in Modern Human Deciduous Molar Enamel Formation Time
Source: Am J Biol Anthropol. 2025 Nov 14;188(3):e70156. doi: 10.1002/ajpa.70156 (PMC12616781; doi:10.1002/ajpa.70156)
Supplement: Supplementary file 4 — Appendix 4 Prenatal formation times. Pairwise comparisons. [file AJPA-188-e70156-s006.pdf]

APPENDIX 4

PRENATAL FORMATION TIMES. Pairwise comparisons

UPPER DM2 Within-samples

Sample 1-Sample 2	Test Statistic	Std. Error	Std. Test Statistic	Sig.	Adj. Sig.[a]
Maori-Canadian	-2.714	7.780	-.349	.727	1.000
Maori-New Zealand	2.882	6.364	.453	.651	1.000
Maori-Pacific	-6.000	8.137	-.737	.461	1.000
Maori-British	15.286	6.596	2.317	.020	.205
Canadian-New Zealand	.168	6.933	.024	.981	1.000
Canadian-Pacific	3.286	8.589	.383	.702	1.000
Canadian-British	12.571	7.147	1.759	.079	.786
New Zealand-Pacific	-3.118	7.331	-.425	.671	1.000
New Zealand-British	12.403	5.572	2.226	.026	.260
Pacific-British	9.286	7.533	1.233	.218	1.000

Each row tests the null hypothesis that the Sample 1 and Sample 2 distributions are the same. Asymptotic significances (2-sided tests) are displayed. The significance level is p=0.050. [a]Significance values have been adjusted by the Bonferroni correction for multiple tests.

LOWER DM2 Within-samples

	Test Statistic	Std. Error	Std. Test Statistic	Sig.	Adj. Sig.[a]
Canadian-Pacific	7.500	8.025	.935	.350	1.000
Canadian-Maori	9.286	8.025	1.157	.247	1.000
Canadian-British	9.750	7.813	1.248	.212	1.000
Canadian-New Zealand	12.550	6.852	1.832	.067	.670
Pacific-Maori	1.786	7.325	.244	.807	1.000
Pacific-British	2.250	7.093	.317	.751	1.000
Pacific-New Zealand	5.050	6.018	.839	.401	1.000
Maori-British	.464	7.093	.065	.948	1.000
Maori-New Zealand	3.264	6.018	.542	.588	1.000
British-New Zealand	-2.800	5.733	-.488	.625	1.000

APPENDIX 4

PRENATAL FORMATION TIMES. Pairwise comparisons

UPPER DM2 Within-samples

	Test Statistic	Std. Error	Std. Test Statistic	Sig.	Adj. Sig.[a]
Iron Age-Medeival	3.318	3.720	.892	.372	1.000
Iron Age-Imperial Rome	9.402	4.756	1.977	.048	.144
Medieval-Imperial Rome	-6.083	4.527	-1.344	.179	.537

LOWER DM2 Within-samples

	Test Statistic	Std. Error	Std. Test Statistic	Sig.	Adj. Sig.[a]
Imperial Rome-Iron Age	-.250	3.767	-.066	.947	1.000
Imperial Rome-Medieval	1.300	3.322	.391	.696	1.000
Iron Age-Medieval	1.050	3.075	.341	.733	1.000

APPENDIX 4

PRENATAL FORMATION TIMES. Pairwise comparisons

UPPER DM1 Within-samples

	Test Statistic	Std. Error	Std. Test Statistic	Sig.	Adj. Sig.[a]
Maori-Canadian	-4.167	4.077	-1.022	.307	1.000
Maori-New Zealand	6.476	3.929	1.648	.099	.596
Maori-British	9.133	4.276	2.136	.033	.196
Canadian-New Zealand	2.310	3.929	.588	.557	1.000
Canadian-British	4.967	4.276	1.161	.245	1.000
New Zealand-British	2.657	4.135	.643	.520	1.000

LOWER DM1 Within-samples

	Test Statistic	Std. Error	Std. Test Statistic	Sig.	Adj. Sig.[a]
Maori-British	.181	4.936	.037	.971	1.000
Maori-Pacific	-2.054	5.149	-.399	.690	1.000
Maori-New Zealand	5.438	5.030	1.081	.280	1.000
British-Pacific	-1.873	4.140	-.452	.651	1.000
British-New Zealand	-5.257	3.992	-1.317	.188	1.000
Pacific-New Zealand	3.384	4.252	.796	.426	1.000

LOWER DM1 Within-samples

	Test Statistic	Std. Error	Std. Test Statistic	Sig.	Adj. Sig.[a]
Iron Age-Imperial Rome	.155	5.166	.030	.976	1.000
Iron Age-Medieval	6.331	5.786	1.094	.274	.822
Imperial Rome-Medieval	6.176	4.387	1.408	.159	.478

UPPER DM1 ARCHAEOLOGICAL MEDIEVAL VS ROMAN (U=0.216; P=0.642)

APPENDIX 4

PRENATAL FORMATION TIMES. Pairwise comparisons

UPPER DM2 Between-samples

	Test Statistic	Std. Error	Std. Test Statistic	Sig.	Adj. Sig.[a]
Iron Age-Medieval	12.391	9.795	1.265	.206	1.000
Iron Age-Maori	17.091	11.090	1.541	.123	1.000
Iron Age-New Zealand	22.473	9.548	2.354	.019	.520
Iron Age-Canadian	22.519	11.930	1.888	.059	1.000
Iron Age-Imperial Rome	24.341	12.523	1.944	.052	1.000
Iron Age-Pacific	28.758	12.523	2.296	.022	.606
**Iron Age-British**	**39.448**	**9.941**	**3.968**	**<.001**	**.002**
Medieval-Maori	4.700	10.403	.452	.651	1.000
Medieval-New Zealand	10.082	8.741	1.154	.249	1.000
Medieval-Canadian	10.129	11.294	.897	.370	1.000
Medieval-Imperial Rome	-11.950	11.919	-1.003	.316	1.000
Medieval-Pacific	16.367	11.919	1.373	.170	1.000
Medieval-British	27.057	9.169	2.951	.003	.089
Maori-New Zealand	5.382	10.171	.529	.597	1.000
Maori-Canadian	-5.429	12.434	-.437	.662	1.000
Maori-Imperial Rome	-7.250	13.004	-.558	.577	1.000
Maori-Pacific	-11.667	13.004	-.897	.370	1.000
Maori-British	22.357	10.542	2.121	.034	.950
New Zealand-Canadian	-.046	11.081	-.004	.997	1.000
New Zealand-Imperial Rome	-1.868	11.717	-.159	.873	1.000
New Zealand-Pacific	-6.284	11.717	-.536	.592	1.000
New Zealand-British	16.975	8.905	1.906	.057	1.000
Canadian-Imperial Rome	-1.821	13.727	-.133	.894	1.000
Canadian-Pacific	6.238	13.727	.454	.650	1.000
Canadian-British	16.929	11.422	1.482	.138	1.000
Imperial Rome-Pacific	4.417	14.246	.310	.757	1.000
Imperial Rome-British	15.107	12.040	1.255	.210	1.000
Pacific-British	10.690	12.040	.888	.375	1.000

APPENDIX 4

PRENATAL FORMATION TIMES. Pairwise comparisons

LOWER DM2 Between-samples

	Test Statistic	Std. Error	Std. Test Statistic	Sig.	Adj. Sig.[a]
Canadian-Imperial Rome	-4.575	12.873	-.355	.722	1.000
Canadian-Iron Age	-5.600	12.137	-.461	.644	1.000
Canadian-Medieval	-8.200	10.511	-.780	.435	1.000
Canadian-Pacific	10.771	11.236	.959	.338	1.000
Canadian-British	12.950	10.940	1.184	.237	1.000
Canadian-Maori	13.057	11.236	1.162	.245	1.000
Canadian-New Zealand	17.025	9.595	1.774	.076	1.000
Imperial Rome-Iron Age	-1.025	12.873	-.080	.937	1.000
Imperial Rome-Medieval	3.625	11.353	.319	.749	1.000
Imperial Rome-Pacific	6.196	12.028	.515	.606	1.000
Imperial Rome-British	8.375	11.751	.713	.476	1.000
Imperial Rome-Maori	8.482	12.028	.705	.481	1.000
Imperial Rome-New Zealand	12.450	10.511	1.185	.236	1.000
Iron Age-Medieval	2.600	10.511	.247	.805	1.000
Iron Age-Pacific	5.171	11.236	.460	.645	1.000
Iron Age-British	7.350	10.940	.672	.502	1.000
Iron Age-Maori	7.457	11.236	.664	.507	1.000
Iron Age-New Zealand	11.425	9.595	1.191	.234	1.000
Medieval-Pacific	2.571	9.457	.272	.786	1.000
Medieval-British	4.750	9.102	.522	.602	1.000
Medieval-Maori	4.857	9.457	.514	.608	1.000
Medieval-New Zealand	8.825	7.432	1.187	.235	1.000
Pacific-British	2.179	9.932	.219	.826	1.000
Pacific-Maori	2.286	10.257	.223	.824	1.000
Pacific-New Zealand	6.254	8.427	.742	.458	1.000
British-Maori	-.107	9.932	-.011	.991	1.000
British-New Zealand	-4.075	8.028	-.508	.612	1.000
Maori-New Zealand	3.968	8.427	.471	.638	1.000

APPENDIX 4

PRENATAL FORMATION TIMES. Pairwise comparisons

UPPER DM1 Between-samples

	Test Statistic	Std. Error	Std. Test Statistic	Sig.	Adj. Sig.[a]
Maori-Imperial Rome	-3.417	6.983	-.489	.625	1.000
Maori-Canadian	-5.583	6.246	-.894	.371	1.000
Maori-New Zealand	7.952	6.019	1.321	.186	1.000
Maori-Medieval	-9.611	5.702	-1.686	.092	1.000
Maori-British	11.467	6.551	1.750	.080	1.000
Imperial Rome-Canadian	2.167	6.983	.310	.756	1.000
Imperial Rome-New Zealand	4.536	6.781	.669	.504	1.000
Imperial Rome-Medieval	6.194	6.501	.953	.341	1.000
Imperial Rome-British	8.050	7.257	1.109	.267	1.000
Canadian-New Zealand	2.369	6.019	.394	.694	1.000
Canadian-Medieval	-4.028	5.702	-.706	.480	1.000
Canadian-British	5.883	6.551	.898	.369	1.000
New Zealand-Medieval	-1.659	5.452	-.304	.761	1.000
New Zealand-British	3.514	6.334	.555	.579	1.000
Medieval-British	1.856	6.034	.308	.758	1.000

APPENDIX 4

PRENATAL FORMATION TIMES. Pairwise comparisons

LOWER DM1 Between-samples

	Test Statistic	Std. Error	Std. Test Statistic	Sig.	Adj. Sig.[a]
Imperial Rome-British	.094	7.885	.012	.990	1.000
Imperial Rome-Iron Age	-1.047	8.656	-.121	.904	1.000
Imperial Rome-Maori	1.261	10.864	.116	.908	1.000
Imperial Rome-Pacific	6.475	8.656	.748	.454	1.000
Imperial Rome-Medieval	11.034	7.351	1.501	.133	1.000
Imperial Rome-New Zealand	14.261	8.231	1.733	.083	1.000
British-Iron Age	-.952	10.106	-.094	.925	1.000
British-Maori	-1.167	12.051	-.097	.923	1.000
British-Pacific	-6.381	10.106	-.631	.528	1.000
British-Medieval	-10.939	9.013	-1.214	.225	1.000
British-New Zealand	-14.167	9.744	-1.454	.146	1.000
Iron Age-Maori	.214	12.569	.017	.986	1.000
Iron Age-Pacific	5.429	10.719	.506	.613	1.000
Iron Age-Medeival	9.987	9.696	1.030	.303	1.000
Iron Age-New Zealand	13.214	10.379	1.273	.203	1.000
Maori-Pacific	-5.214	12.569	-.415	.678	1.000
Maori-Medeival	-9.773	11.709	-.835	.404	1.000
Maori-New Zealand	13.000	12.280	1.059	.290	1.000
Pacific-Medieval	-4.558	9.696	-.470	.638	1.000
Pacific-New Zealand	7.786	10.379	.750	.453	1.000
Medieval-New Zealand	3.227	9.318	.346	.729	1.000
