# Supplementary material for: Variation in Modern Human Deciduous Molar Enamel Formation Time
Source: Am J Biol Anthropol. 2025 Nov 14;188(3):e70156. doi: 10.1002/ajpa.70156 (PMC12616781; doi:10.1002/ajpa.70156)
Supplement: Supplementary file 5 — Appendix 5 Extension rates for DM1. [file AJPA-188-e70156-s002.pdf]

APPENDIX 5

EXTENSION RATES FOR DM1

Mean Extension Rate (sd) for segments of the dm1 EDJ in µm/day

Segment	British dm[1] (*n*=6)	NZ Eur dm[1] (*n*=16)	Maori dm[1] (*n*=10)	Pacific dm[1] (*n*=)	Medieval dm[1] (n=10)	Roman dm[1] (n=5)	Iron Age dm[1] (n=5)
1	28.99 (6.00)	33.56 (7.76)	32.46 (5.28)	-	36.69 (2.64)	35.16 (4.00)	39.53 (5.49)
	23.24 (1.41)	29.38 (7.57)	26.06 (5.10)	-	29.60 (2.93)	28.74 (6.65)	32.14 (5.16)
2	17.75 (1.63)	23.07 (5.86)	17.23 (2.20)	-	20.37 (2.88)	20.81 (3.84)	22.33 (3.81)
	14.93 (2.02)	18.63 (4.42)	15.41 (3.56)	-	16.72 (2.20)	16.56 (2.46)	22.06 (4.60)
3	13.19 (1.60)	15.26 (3.62)	12.63 (2.09)	-	12.11 (2.15)	15.49 (3.94)	17.42 (1.85)
	11.25 (1.85)	13.36 (3.64)	12.16 (1.80)	-	11.27 (1.22)	13.75 (4.63)	17.52 (1.55)
4	9.64 (1.01)	11.07 (2.31)	10.47 (1.56)	-	10.15 (0.72)	9.71 (1.84)	14.30 (1.61)
	8.11 (1.04)	7.38 (0.91)	9.89 (1.48)		11.53 (1.37)	9.91 (1.64)	13.37 (1.38)

Segment	British dm_1 (*n*=10)	NZ Eur dm_1 (*n*=8)	Maori dm_1 (*n*=10)	Pacific Islander dm_1 (*n*=7)	Medieval dm_1 (n=10)	Roman dm_1 (n=20)	Iron Age dm_1 (n=5)
1	25.90 (8.26)	31.24 (5.60)	28.26 (5.59)	37.40 (3.54)	35.89 (4.67)	34.68 (4.01)	36.93 (5.69)
	22.86 (7.67)	26.55 (5.08)	23.85 (3.77)	30.53 (3.81)	30.82 (4.60)	26.69 (4.51)	30.55 (3.39)
2	18.71 (2.81)	20.73 (6.59)	19.35 (2.18)	19.21 (1.01)	20.58 (4.73)	19.98 (2.28)	22.93 (2.81)
	16.28 (3.70)	16.44 (4.55)	15.02 (1.85)	16.86 (1.83)	17.57 (3.97(	17.05 (3.70)	20.27 (3.12)
3	12.06 (3.07)	14.86 (3.17)	13.84 (2.01)	15.44 (2.47)	13.24 (2.32)	15.71 (3.73)	16.43 (2.26)
	12.16 (2.69)	8.75 (1.05)	12.36 (1.10)	12.42 (1.58)	13.79 (2.64)	15.42 (2.87)	14.00 (3.20)
4	10.13 (1.70)	9.24 (2.60)	12.40 (2.60)	12.11 (2.51)	14.77 (2.41)	13.85 (2.50	13.91 (2.52)
	10.31 (1.09)	9.01 (2.45)	11.65 (2.33)	11.77 (1.06)	11.13 (1.27)	11.74 (1.71)	10.83 (2.09)
