# Supplementary material for: Variation in Modern Human Deciduous Molar Enamel Formation Time
Source: Am J Biol Anthropol. 2025 Nov 14;188(3):e70156. doi: 10.1002/ajpa.70156 (PMC12616781; doi:10.1002/ajpa.70156)
Supplement: Supplementary file 6 — Appendix 6 Extension rates for DM2. [file AJPA-188-e70156-s001.pdf]

APPENDIX 6

EXTENSION RATES FOR DM2

Mean Extension Rate (sd) for segments of the dm2 EDJ in µm/day

Segment	British dm^2 (n=15)	NZ Eur dm^2 (n=12)	Maori dm^2 (n=8)	Pacific dm^2 (n=8)	Medieval dm^2 (n=8)	Roman dm^2 (n=5)	Iron Age dm^2 (n=5)
1	36.11 (4.81)	35.01 (6.72)	33.17 (4.89)	38.89 (3.59)	40.61 (1.78)	37.55 (1.62)	42.84 (3.12)
	31.30 (3.96)	29.86 (5.96)	26.56 (4.56)	33.91 (5.01)	34.35 (2.58)	30.27 (1.88)	35.47 (3.00)
2	21.43 (3.90)	19.47 (5.68)	16.26 (2.27)	19.07 (3.27)	20.95 (2.86)	18.81 (1.99)	26.53 (2.65)
	16.79 (3.31)	17.11 (3.61)	14.06 (3.70)	15.34 (1.54)	16.85 (2.71)	18.21 (0.69)	21.05 (2.02)
3	13.53 (3.12)	12.41 (3.82)	11.86 (2.35)	12.67 (2.36)	14.03 (1.55)	13.34 (1.86)	18.60 (1.59)
	12.78 (1.67)	10.60 (1.55)	11.49 (1.28)	11.77 (1.29)	12.07 (1.98)	12.90 (1.52)	16.03 (3.29)
4	10.86 (1.66)	9.91 (2.99)	9.65 (1.86)	8.77 (0.95)	10.45 (1.63)	9.65 (1.520	12.92(2.07)
	9.66 (1.79)	7.38 (1.63)	9.89 (0.94)	7.45 (1.05)	10.84 (1.58)	7.05 (0.85)	9.66 (1.05)

	British dm$_2$ (n=15)	NZ Eur dm$_2$ (n=15)	Maori dm$_2$ (n=11)	Pacific Islander dm$_2$ (n=8)	Medieval dm$_2$ (n=8)	Imperial (n=5)	Iron Age (n=5)
1	33.57 (7.38)	33.38 (6.95)	29.11 (5.63)	34.86 (5.50)	40.46 (2.89)	37.38 (2.66)	38.36 (3.52)
	28.11 (6.30)	27.65 (6.86)	22.86 (4.77)	28.52 (4.13)	32.62 (2.08)	28.75 (2.94)	28.71 (3.33)
2	17.92 (3.56)	17.11 (2.26)	15.55 (3.65)	17.58 (2.63)	20.42 (2.53)	17.63 (1.87)	22.11 (3.46)
	14.10 (3.51)	14.04 (2.05)	13.41 (2.79)	15.34 (2.60)	17.51 (2.38)	18.77 (2.37)	20.24 (2.75)
3	12.78 (3.34)	12.38 (1.41)	10.90 (1.83)	13.46 (1.89	13.96 (2.02)	14.65 (2.05)	14.88 (1.95)
	11.77 (2.58)	10.63 (1.06)	9.98 (2.32)	12.31 (2.04)	12.26 (1.25)	11.66 (0.30)	14.13 (1.61)
4	11.15 (2.19)	11.28 (1.84)	9.00 (2.75)	11.59 (1.01)	11.55 (1.39)	12.54 (0.82)	15.56 (1.5)
	9.97 (1.01)	8.96 (0.95)	9.88 (1.34)	9.57 (0.82)	10.56 (1.70)	8.22 (0.83)	9.80 (0.90)
