# Supplementary material for: Variation in Modern Human Deciduous Molar Enamel Formation Time
Source: Am J Biol Anthropol. 2025 Nov 14;188(3):e70156. doi: 10.1002/ajpa.70156 (PMC12616781; doi:10.1002/ajpa.70156)
Supplement: Supplementary file 7 — Appendix 7 Average enamel thickness. Pairwise comparisons. [file AJPA-188-e70156-s004.pdf]

APPENDIX 7.
Average Enamel Thickness. Pairwise comparisons

WITHIN SAMPLES

Archaeological - Upper dm2 Pairwise Comparisons

Sample 1-Sample 2	Test Statistic	Std. Error	Std. Test Statistic	Sig.	Adj. Sig.[a]
Medieval-Roman	-1.116	5.876	-.190	.849	1.000
Medieval-Iron	-4.191	3.967	-1.056	.291	.872
Roman-Iron	3.075	5.990	.513	.608	1.000

Each row tests the null hypothesis that the Sample 1 and Sample 2 distributions are the same. Asymptotic significances (2-sided tests) are displayed. The significance level is .050. [a]Significance values have been adjusted by the Bonferroni correction for multiple tests.

Archaeological - Lower dm2 Pairwise Comparisons

Sample 1-Sample 2	Test Statistic	Std. Error	Std. Test Statistic	Sig.	Adj. Sig.[a]
Iron-Medieval	2.810	4.610	.609	.542	1.000
Iron-Roman	-10.190	5.540	-1.839	.066	.198
Medieval-Roman	-7.381	4.346	-1.698	.089	.268

Present-day - Upper dm2 Pairwise Comparisons

Sample 1-Sample 2	Test Statistic	Std. Error	Std. Test Statistic	Sig.	Adj. Sig.[a]
Maori-Pacific	-3.993	9.602	-.416	.678	1.000
Maori-NZ-European	6.959	7.380	.943	.346	1.000
Maori-Canadian	10.017	10.062	.996	.319	1.000
Maori-British	10.040	7.486	1.341	.180	1.000
Pacific-NZ-European	2.966	8.411	.353	.724	1.000
Pacific-Canadian	6.024	10.840	.556	.578	1.000
Pacific-British	6.048	8.504	.711	.477	1.000
NZ-European-Canadian	-3.058	8.932	-.342	.732	1.000
NZ-European-British	-3.082	5.881	-.524	.600	1.000
Canadian-British	.024	9.020	.003	.998	1.000

APPENDIX 7.
Average Enamel Thickness. Pairwise comparisons

WITHIN SAMPLES

Present-day - Lower dm2 Pairwise Comparisons

Sample 1-Sample 2	Test Statistic	Std. Error	Std. Test Statistic	Sig.	Adj. Sig.[a]
Pacific-NZ-European	6.008	6.879	.873	.382	1.000
Pacific-Maori	6.571	8.255	.796	.426	1.000
Pacific-Canadian	6.911	7.993	.865	.387	1.000
Pacific-British	9.516	7.240	1.314	.189	1.000
NZ-European-Maori	-.563	6.879	-.082	.935	1.000
NZ-European-Canadian	-.903	6.562	-.138	.891	1.000
NZ-European-British	-3.509	5.621	-.624	.533	1.000
Maori-Canadian	.339	7.993	.042	.966	1.000
Maori-British	2.945	7.240	.407	.684	1.000
Canadian-British	2.606	6.940	.375	.707	1.000

Archaeolgical - Lower dm1 Pairwise Comparisons

Sample 1-Sample 2	Test Statistic	Std. Error	Std. Test Statistic	Sig.	Adj. Sig.[a]
Medieval-Roman	-6.630	4.759	-1.393	.164	.491
Medieval-Iron	-7.650	5.993	-1.277	.202	.605
Roman-Iron	1.020	5.562	.183	.855	1.000

Archaeolgical - Upper dm1

Medieval v Roman	
Test Statistic	.011
Degree Of Freedom	1
Asymptotic Sig.(2-sided test)	.915

Present-day - Lower dm1 Pairwise Comparisons

Sample 1-Sample 2	Test Statistic	Std. Error	Std. Test Statistic	Sig.	Adj. Sig.[a]
Maori-NZ-European	5.467	6.186	.884	.377	1.000
Maori-Pacific	-8.314	7.014	-1.185	.236	1.000
Maori-British	9.314	6.241	1.492	.136	.814
NZ-European-Pacific	-2.848	5.483	-.519	.604	1.000
NZ-European-British	-3.848	4.452	-.864	.387	1.000

APPENDIX 7.
Average Enamel Thickness. Pairwise comparisons

Pacific-British	1.000	5.545	.180	.857	1.000

WITHIN SAMPLES

Present-day - Upper dm1 Pairwise Comparisons

Sample 1-Sample 2	Test Statistic	Std. Error	Std. Test Statistic	Sig.	Adj. Sig.[a]
Canadian-Maori	-11.058	6.010	-1.840	.066	.395
Canadian-British	11.841	5.414	2.187	.029	.172
Canadian-NZ-European	13.035	5.853	2.227	.026	.156
Maori-British	.783	5.833	.134	.893	1.000
Maori-NZ-European	1.977	6.243	.317	.751	1.000
British-NZ-European	1.194	5.672	.211	.833	1.000

APPENDIX 7.
Average Enamel Thickness. Pairwise comparisons

BETWEEN SAMPLES

Lower dm2 - Pairwise Comparisons of Populations

Sample 1-Sample 2	Test Statistic	Std. Error	Std. Test Statistic	Sig.	Adj. Sig.[a]
Iron-Pacific	10.024	14.053	.713	.476	1.000
Iron-NZ-European	19.444	11.907	1.633	.102	1.000
Iron-Canadian	20.667	13.641	1.515	.130	1.000
Iron-Maori	20.881	14.053	1.486	.137	1.000
Iron-British	25.859	12.466	2.074	.038	1.000
Medieval-Pacific	2.714	11.024	.246	.806	1.000
Medieval-NZ-European	12.135	8.113	1.496	.135	1.000
Medieval-Canadian	13.357	10.494	1.273	.203	1.000
Medieval-Maori	13.571	11.024	1.231	.218	1.000
Medieval-British	18.549	8.914	2.081	.037	1.000
Pacific-Roman	-16.429	13.501	-1.217	.224	1.000
NZ-European-Roman	-7.008	11.251	-.623	.533	1.000
Canadian-Roman	-5.786	13.073	-.443	.658	1.000
Maori-Roman	-5.571	13.501	-.413	.680	1.000
British-Roman	-.593	11.841	-.050	.960	1.000

Each row tests the null hypothesis that the Sample 1 and Sample 2 distributions are the same.Asymptotic significances (2-sided tests) are displayed. The significance level is .050. [a]Significance values have been adjusted by the Bonferroni correction for multiple tests.

APPENDIX 7.
Average Enamel Thickness. Pairwise comparisons

BETWEEN SAMPLES

Upper dm2 - Pairwise Comparisons of Population

Sample 1-Sample 2	Test Statistic	Std. Error	Std. Test Statistic	Sig.	Adj. Sig.[a]
Medieval-Maori	10.058	12.124	.830	.407	1.000
Medieval-Pacific	15.801	13.720	1.152	.249	1.000
Medieval-NZ-European	19.419	9.620	2.019	.044	1.000
Medieval-Canadian	23.491	14.532	1.617	.106	1.000
Medieval-British	25.944	9.825	2.640	.008	.232
Roman-Maori	7.800	16.997	.459	.646	1.000
Roman-Pacific	13.543	18.170	.745	.456	1.000
Roman-NZ-European	17.161	15.312	1.121	.262	1.000
Roman-Canadian	21.233	18.791	1.130	.258	1.000
Roman-British	23.686	15.442	1.534	.125	1.000
Iron-Maori	3.400	12.509	.272	.786	1.000
Iron-Pacific	9.143	14.063	.650	.516	1.000
Iron-NZ-European	12.761	10.102	1.263	.207	1.000
Iron-Canadian	16.833	14.855	1.133	.257	1.000
Iron-British	19.286	10.298	1.873	.061	1.000

APPENDIX 7.
Average Enamel Thickness. Pairwise comparisons

BETWEEN SAMPLES

Lower dm1 - Pairwise Comparisons of Population

Sample 1-Sample 2	Test Statistic	Std. Error	Std. Test Statistic	Sig.	Adj. Sig.[a]
Maori-Medieval	-8.938	13.681	-.653	.514	1.000
Maori-Roman	-21.080	13.081	-1.611	.107	1.000
Maori-Iron	-22.300	14.625	-1.525	.127	1.000
Medieval-NZ-European	6.396	9.597	.666	.505	1.000
Medieval-Pacific	13.634	12.100	1.127	.260	1.000
Medieval-British	14.563	9.772	1.490	.136	1.000
NZ-European-Roman	-5.747	8.721	-.659	.510	1.000
NZ-European-Iron	-6.967	10.901	-.639	.523	1.000
Roman-Pacific	1.491	11.418	.131	.896	1.000
Roman-British	2.420	8.913	.272	.786	1.000
Iron-Pacific	.271	13.159	.021	.984	1.000
Iron-British	1.200	11.056	.109	.914	1.000

Upper dm1 - Pairwise Comparisons of Population

Sample 1-Sample 2	Test Statistic	Std. Error	Std. Test Statistic	Sig.	Adj. Sig.[a]
Canadian-Roman	-7.740	9.923	-.780	.435	1.000
Canadian-Medieval	-8.905	7.948	-1.120	.263	1.000
Roman-Maori	8.625	10.475	.823	.410	1.000
Roman-British	10.208	9.668	1.056	.291	1.000
Roman-NZ-European	12.148	10.261	1.184	.236	1.000
Medieval-Maori	7.461	8.627	.865	.387	1.000
Medieval-British	9.044	7.627	1.186	.236	1.000
Medieval-NZ-European	10.983	8.366	1.313	.189	1.000
