# Supplementary material for: Variation in Modern Human Deciduous Molar Enamel Formation Time
Source: Am J Biol Anthropol. 2025 Nov 14;188(3):e70156. doi: 10.1002/ajpa.70156 (PMC12616781; doi:10.1002/ajpa.70156)
Supplement: Supplementary file 8 — Appendix 8 Test of lateral enamel scaling proportions. [file AJPA-188-e70156-s005.pdf]

APPENDIX 8

TEST OF LATERAL ENAMEL SCALING PROPORTIONS

Table S1. Percentage of permanent first molar lateral enamel formation time.

	Outer Lateral Enamel			EDJ
	Perikymata	Perikymata	Retzius lines	Extension rates
	Modesto-Mata et al. 2020[1]	Guatelli-Steinberg & Reid 2008[2]	Reid and Dean 2006[3]	This study
UM1				
25%	15	15	14	8
50%	16	16	17	16
75%	23	29	28	31
100%	46	40	40	44
LM1				
25%	17	14	15	9
50%	18	18	18	18
75%	26	30	31	31
100%	39	38	37	42

[1]n = 6, taken from their Figure S4.

[2]n = 27, their Figures 6 & 7. Estimate derived from number of days calculated from the number of PK per decile, reworked into quarters, and multiplied by a periodicity of 8.5 days (based on Fig 4, Guatelli-Steinberg & Reid 2008; a similar value was suggested in Modesto-Mata et al. 2020).

[3]calculated directly from the number of days to form each decile (their Table 5, UM1 protocone & LM1 protoconid). They report cumulative days for each decile so differences between each decile were calculated to arrive at the actual number per decile. Cuspal formation time was not included. **See Table S2 and S3 below for calculations**.

APPENDIX 8

TEST OF LATERAL ENAMEL SCALING PROPORTIONS

Table S2. Perikymata calculations.

Source	Tooth	Increments	PK count	Days	% of total time
Modesto-Mata et al. 2020	UM1	25%	16	136	15
		50%	17	145	16
		75%	25	213	23
		100%	49	417	46
	LM1	25%	17	145	17
		50%	19	162	18
		75%	27	230	26
		100%	40	340	39
Guatelli-Steinberg & Reid 2008	UM1	25%	13	111	15
		50%	14	119	16
		75%	25	213	29
		100%	35	298	40
	LM1	25%	12	102	14
		50%	16	136	18
		75%	26	221	30
		100%	33	281	38

APPENDIX 8

TEST OF LATERAL ENAMEL SCALING PROPORTIONS

Table S3. Retzius lines calculations. Reid and Dean (2006) values were calculated directly from the number of days to form each decile (Table 5, UM1 protocone & LM1 protoconid). They report cumulative days for each decile so differences between each decile were calculated to arrive at the actual number per decile. Cuspal formation time was not included.

Source	Tooth	Increments	Days	% of total time
Reid and Dean 2006	UM1	25%	103.5	14
		50%	128.5	17
		75%	209	28
		100%	295	40
	LM1	25%	106.5	15
		50%	131.5	18
		75%	221	31
		100%	265	37
